# Unraveling the effector mechanism of citrulline on sow lactation and offspring growth: an integrative multi-omics analysis

**DOI:** 10.1186/s40104-026-01414-x

**Published:** 2026-06-16

**Authors:** Yating Chen, Shenglan Hu, Yiwen Ji, Fang Gu, Chenyang Zhang, Kaiguo Gao, Xiaolu Wen, Li Wang, Xiaoli Dong, Jisoo Tak, Hao Xiao, Yaoyao Xia

**Affiliations:** 1 College of Animal Science and Technology, Southwest University, Chongqing, 400715 China; 2 State Key Laboratory of Swine and Poultry Breeding; Key Laboratory of Animal Nutrition and Feed Science in South China, Ministry of Agriculture and Rural Affairs; Guangdong Provincial Key Laboratory of Animal Breeding and Nutrition; Institute of Animal Science, Guangdong Academy of Agricultural Sciences, Guangzhou, 510640 China; 3 CJ International Trading Co., Ltd., Seoul, South Korea; 4 CJ Cheiljedang, Seoul, South Korea

**Keywords:** Citrulline, Intestinal homeostasis, Lactation performance, Metabolome–microbiome

## Abstract

**Background:**

Citrulline (Cit), an effective precursor of arginine (Arg), escapes hepatic catabolism to be almost completely absorbed into the systemic circulation, thereby being efficiently converted to Arg in the kidneys to enhance its systemic bioavailability. This study investigated the effects of dietary Cit supplementation on lactation performance in sows, as well as the underlying mechanisms related to intestinal health in their suckling piglets, using multi-omics analyses.

**Results:**

Dietary Arg and Cit supplementation significantly increased average daily feed intake of lactating sows. Milk fat content and plasma nitric oxide (NO) concentration increased significantly in the Arg group and the 40%Cit group (*P* < 0.05), while milk threonine content increased slightly (*P* = 0.084). Consequently, the average daily gain of suckling piglets over the 21-day lactation period was also significantly improved. Furthermore, maternal 40%Cit supplementation improved the intestinal health of offspring by enhancing jejunal morphology and upregulating the expression of the tight junction protein occludin (*P* < 0.05), indicating a strengthened intestinal barrier. Mechanistically, this was achieved by activating the mTOR/S6 pathway in the piglets' jejunum. Maternal 40%Cit supplementation upregulated the expression of proteins related to mitochondrial fusion and fission (MFN2 and MFF, *P* < 0.05), and the protein expression of OPA1 showed an increasing trend (*P* = 0.097), indicating the structural and functional status of mitochondria was improved. Maternal 40%Cit supplementation also modulated the gut microbiota of piglets, increasing the abundance of beneficial bacteria (*Lachnoclostridium*). Metabolomic analysis of sow milk identified 58 differential metabolites. Among these metabolites, palmitic acid levels were significantly increased and positively correlated with the abundance of *Lachnoclostridium* in the intestine (*P* < 0.05).

**Conclusions:**

Dietary Cit supplementation enhanced sow lactation performance and improved intestinal barrier function in their offspring via activation of the jejunal mTOR/S6 pathway and improved mitochondrial structure and function in the piglet jejunum. These benefits were further supported by modulation of the gut microbiota and alterations in the milk fat and metabolome, ultimately promoting piglet growth.

**Graphical Abstract:**

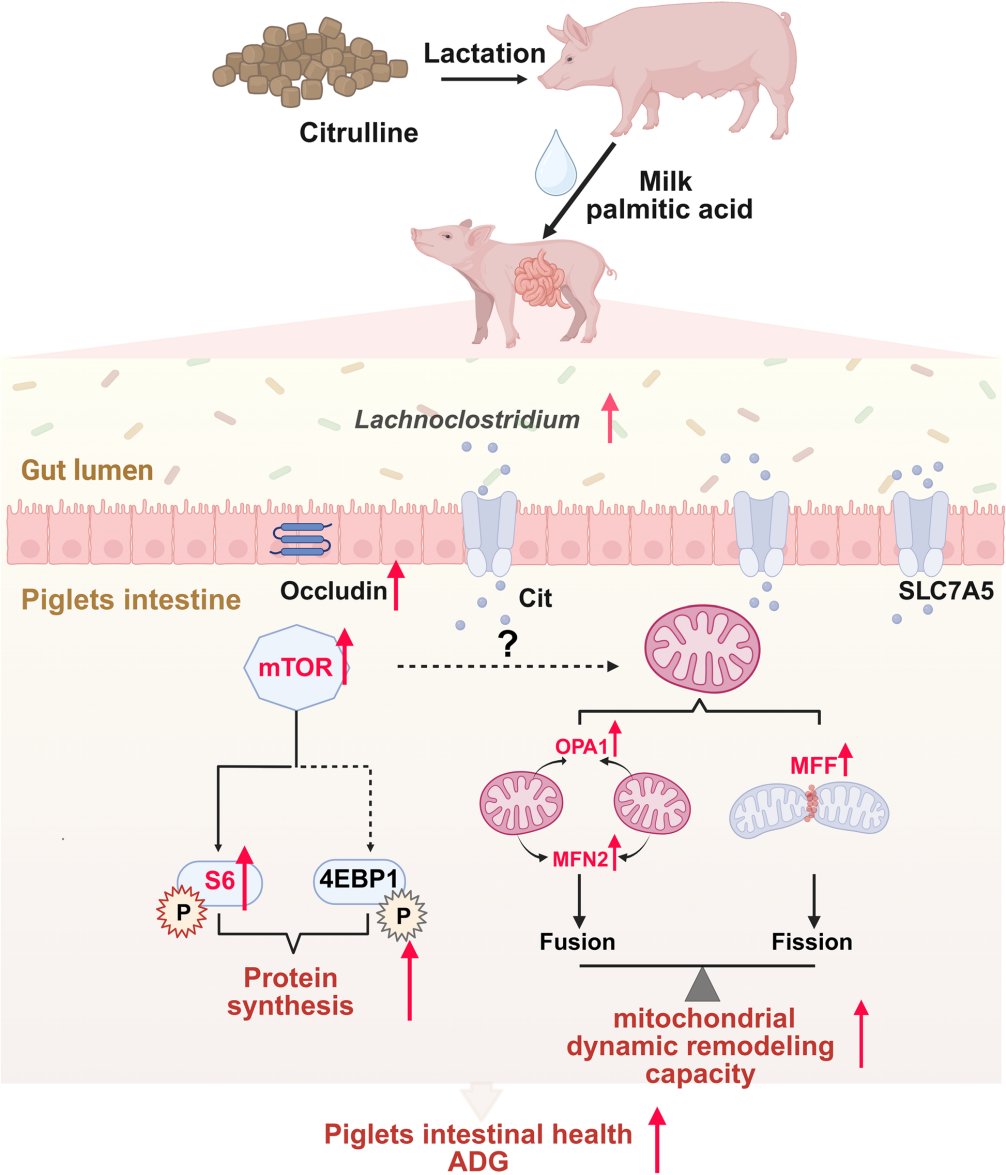

**Supplementary Information:**

The online version contains supplementary material available at 10.1186/s40104-026-01414-x.

## Introduction

Lactation performance in sows is a critical determinant of productivity and economic sustainability in the pig industry, with nutritional restriction being a major limiting factor. Functional amino acids such as arginine (Arg) have been incorporated into sow diets to improve reproductive outcomes. Supplementing gestating sows with 1.0% Arg has been shown to increase placental weight, total umbilical cord diameter of live-born littermates, total number of piglets born, number born alive, and litter weight [1, 2]. In lactating sows, similar Arg supplementation improves milk concentrations of polyamines, free amino acids, and milk fat, enhances suckling piglet weight gain [3], and increases bioactive compounds such as Arg metabolites in milk, thereby promoting intestinal development in piglets [4]. Although substantial evidence confirms that dietary Arg at 0.8%−1.5% improves sow reproductive performance [3, 5–7], its efficacy is constrained by extensive first-pass metabolism in the liver, limiting systemic bioavailability. This has sparked interest in citrulline (Cit), a natural and highly bioavailable precursor of Arg that bypasses hepatic first-pass metabolism and more effectively elevates systemic Arg levels. Indeed, oral Cit administration results in a more pronounced and sustained increase in plasma Arg concentrations compared with Arg alone [8]. However, whether Cit supplementation exerts similar beneficial effects on lactating sows remains unknown.

Studies have shown that administration of Cit (155 μmol/kg) by injection during gestation can increase the number of follicles in ewes [9], while dietary supplementation with Cit (10 g/d) improves the kidding rate and twinning rate by 15.90% and 40.21%, respectively [10]. Intraperitoneal injection of Cit solution (155 μmol/kg) during proestrus also enhances reproductive performance in primiparous and multiparous dairy cows [11]. Previous studies suggest that Cit supplementation can improve milk yield and composition, enhance immune and antioxidant capacity, and maintain vascular function in Hu sheep [12]. Furthermore, dietary supplementation with 1% Cit in lactating sows under heat stress during summer has been found to reduce respiratory rate of sows and pre-weaning mortality of suckling piglets [13]. However, the effects of dietary Cit supplementation during lactation on sow productive performance and suckling piglet intestinal health, as well as the underlying mechanisms, remain unexplored under normal physiological conditions.

Therefore, this study aimed to investigate the effects of dietary Cit supplementation during lactation on the productive performance of sows, and the potential mechanisms by which maternal Cit supplementation improves intestinal health in suckling piglets using integrated multi-omics analysis of milk metabolites and fecal microbiome and molecular biology analyses.

## Materials and methods

### Animal ethics statement

All animal procedures involved in these experiments were approved by the Animal Care Committee of the Institute of Animal Science, Guangdong Academy of Agricultural Sciences (Guangzhou, P.R. China). The approval number for these procedures is GAASISA-2024-028.

### Experimental design, animals, and diet

This experiment was conducted at the Guangzhou Baiyun experimental base. A total of 150 purebred Landrace lactating sows with similar parity (5.42 ± 1.43), backfat thickness (14.83 ± 3.21 mm), and body weight (245.11 ± 30.27 kg) were randomly assigned to five dietary groups: a control diet (Con group, with no Arg supplementation); a diet supplemented with 0.6% Arg (98.5% purity; the Arg group); and three control diets supplemented with Cit (CJ Cheiljedang, 99.0% purity). Cit was supplemented to replace 20%, 30%, or 40% of the Arg on an isonitrogenous basis (designated the 20%Cit, 30%Cit or 40%Cit groups, respectively). All diets were made isonitrogenous by the addition of L-alanine. The level of supplemental Arg was determined based on multiple relevant studies in this field [3, 5–7]. To date, no studies have reported on the effect of varying the amount of Cit replacing Arg in diets for lactating sows. Each diet was considered a separate treatment group, with 30 sows in each group. On the day before the due date, administer 1 mL of cloprostenol-induced synchronized parturition via intramuscular injection and await farrowing. If the piglet remains retained and the sow exhibits respiratory distress, perform assisted farrowing. The individual BW of the neonatal piglets was measured on d 3 of lactation. Litters were adjusted according to similar BW and good health, and were cross-fostered among sows to ensure uniformity, with each sow nursing 12 piglets. The experiment then began and lasted for 18 d. The diet for gestating sows is primarily based on corn for energy and rapeseed meal for protein, whereas the basal diet for lactating sows is corn‑soybean meal‑based. All sows were maintained under consistent and stable environmental conditions (temperature: 26.19 ± 0.42°C; relative humidity: 62%−68%) and had free access to feed and water during lactation. To ensure experimental accuracy, no creep feed was provided to suckling piglets. Feed intake of sows was recorded daily. Sow-piglet health assessments (mental state, skin lesions and diarrhea) were monitored and recorded daily.

### Dietary analysis

The basal diet was formulated to meet or exceed the nutrient requirements for pregnant and lactating sows as recommended by the NRC (2012) [14]. The diets for all test sows during gestation were kept consistent, and the ingredient composition and nutritional levels of the basal diet during lactation are presented in Table 1. The crude protein, crude fat (ether extract), calcium and total phosphorus contents of the feed were analyzed using the methods set out in the Chinese National Standards GB/T 6432–2018 [15], GB/T 6433–2025 [16], GB/T 6436–2018 [17], and GB/T 6437–2018 [18], respectively.

**Table 1:** Ingredients and nutrient levels of experimental diets for sows (as-fed basis)^1^

Item	Con	Arg	20%Cit	30%Cit	40%Cit
Ingredients, %					
Corn (CP 8%)	57.32	59.95	57.32	57.36	57.40
Wheat flour	9.00	8.00	9.00	9.00	9.00
Soybean meal (CP 44%)	17.58	15.95	17.58	17.57	17.57
Fat powder	2.03	2.15	2.20	2.25	2.30
Rapeseed meal	8.00	8.00	8.00	8.00	8.00
L-Lysine HCl	0.45	0.50	0.45	0.45	0.45
DL-Methionine	0.11	0.12	0.11	0.11	0.11
L-Threonine	0.18	0.19	0.18	0.18	0.18
L-Tryptophan	0.05	0.05	0.05	0.05	0.05
L-Valine	0.26	0.29	0.26	0.26	0.26
L-Arginine	-	0.60	-	-	-
L-Citrulline	-	-	0.12	0.18	0.24
L-Alanine	0.88	-	0.59	0.45	0.30
L-Isoleucine	0.02	0.05	0.02	0.02	0.02
L-Histidine	0.01	0.02	0.01	0.01	0.01
NaCl	0.40	0.40	0.40	0.40	0.40
Limestone	1.41	1.43	1.41	1.41	1.41
CaHPO_4_	1.15	1.15	1.15	1.15	1.15
Choline chloride	0.15	0.15	0.15	0.15	0.15
Premix^2^	1.00	1.00	1.00	1.00	1.00
Total	100.00	100.00	100.00	100.00	100.00
Nutrient levels^3^					
NE, Mcal/kg	2.52	2.53	2.53	2.53	2.52
CP, %	17.02	16.89	17.21	16.94	16.87
CEE, %	3.67	3.75	3.61	3.96	3.53
Ca, %	0.99	1.00	0.92	1.04	0.97
P, %	0.59	0.64	0.59	0.59	0.62
SID N, %	2.48	2.52	2.47	2.47	2.47
SID Lys, %	1.05	1.05	1.05	1.05	1.05
SID Arg, %	0.90	1.45	0.90	0.90	0.90
Arg/Lys	0.86	1.38	0.86	0.86	0.86
SID Thr, %	0.78	0.77	0.78	0.78	0.78
SID Trp, %	0.19	0.19	0.19	0.19	0.19
SID Val, %	0.91	0.91	0.91	0.91	0.91
SID His, %	0.38	0.38	0.38	0.38	0.38
SID Met, %	0.33	0.33	0.33	0.33	0.33
SID Ile, %	0.58	0.58	0.58	0.58	0.58
SID Leu, %	1.18	1.15	1.18	1.18	1.18

*NE* Net energy, *CP* Crude protein, *CEE* Crude ether extract, *SID* Standardized ileal digestibility

^1^*Con* Control, *Arg* Diet with arginine, *20%Cit* Diet with 20% Arg replaced by citrulline, *30%Cit* Diet with 30% Arg replaced by citrulline, *40%Cit* Diet with 40% Arg replaced by citrulline

^2^The premix provided the following per kg of the diet during lactation: Cu, 2,000 mg; Fe, 12,000 mg; Zn, 9,000 mg; Mn, 5,000 mg; I, 50 mg; Se, 42 mg; Co, 50 mg; Cr, 18.5 mg; VA, 720,000 IU; VD, 323,000 IU; VE, 12,600 mg; VK, 920 mg; VB_1_, 380 mg; VB_2_, 840 mg; VB_6_, 520 mg; VB_12_, 5.6 μg; folic acid, 570 mg; pantothenic acid, 4,490 mg; niacin, 4,940 mg; and biotin 290 mg

^3^All nutrient levels are measured values, except for SID AA, SID N and NE which are calculated value (NRC, 2012) [14]

### Data and sample collection

Average daily feed intake (ADFI) was calculated by dividing the total feed intake during lactation by the total number of experimental days. Sow backfat thickness was measured at farrowing and on d 21 of lactation, using the methodology detailed in our previous study [19]. Litter weight was measured on d 3 and 21 of lactation. On d 21 of lactation, 10 randomly selected sows per group (representative of ADFI, BW loss and weaning litter weight on d 21 of lactation, Table S1) were selected for sampling. Blood samples were collected from the anterior vena cava of the sows at 08:00 after an overnight fast using heparin sodium anticoagulant tubes. These samples were then centrifuged at 1,000 × *g* for 10 min to isolate the plasma. Milk samples were collected using the standardized procedure described previously [19]. Before milk collection, piglets were temporarily separated from sows. Each sow was then injected with 2 mL of oxytocin via the marginal ear vein, followed by collecting milk samples using sterile centrifuge tubes. Fresh feces samples were also collected from sows on lactation d 21 using sterile tubes and promptly frozen at −80 °C. The evening before the end of the trial, the piglets were separated from the sows at 20:00 and weighed at 08:00 the following morning. In line with the principle of selecting piglets with BW close to the litter average and excluding those with extremely low or high BW, 6 representative litters per group were randomly chosen from the sampled sows. One castrated male piglet was randomly selected to euthanize from each litter for sampling (*n* = 6/group; Table S2). Segments of the jejunum, ileum and colon were harvested. Middle sections (3–5 cm in length) of the jejunal and ileal tissues were preserved at −80 °C. Meanwhile, additional jejunal and ileal segments were opened longitudinally along the mesenteric border and the mucosal layer carefully scraped off using a sterile glass slide or scalpel blade. The scrapings were then transferred immediately to cryotubes and stored at −80 °C for further analysis.

### Hormone analyses

The concentrations of estradiol (Cat. No. MM-048001), insulin (Cat. No. MM-039001), prolactin (Cat. No. MM-0907O1) and progesterone (Cat. No. MM-1205501) in sow plasma were determined using commercial enzyme-linked immunosorbent assay (ELISA) kits (Meimian Industrial Co., Ltd., Jiangsu, China). Estradiol was detected with an LOD of < 0.1 pg/mL and an inter-assay CV of < 15%. Insulin was detected with an LOD of < 1 mU/L, an intra-assay CV of < 15%, and an inter-assay CV of < 15%. Prolactin was detected with an LOD ranging from 0.094 to 1.0 ng/mL, with an intra-assay CV of < 10% and an inter-assay CV of < 15%. Progesterone was detected with an LOD of 0.05 ng/mL, an intra-assay CV of < 8% and an inter-assay CV of < 12%. Nitric oxide (NO) levels in plasma were measured using a colorimetric assay kit (A012-1, Nanjing Jiancheng Bioengineering Institute, Nanjing, China) in accordance with the manufacturer's instructions.

### Intestinal digestive enzyme activity

The activities of lipase (LPS, Cat. No. A054-2-1), trypsin (Cat. No. A080-2-2) and amylase (Cat. No. C016-1-2) in the jejunal mucosa of suckling piglets were determined using commercial assay kits (Nanjing Jiancheng Bioengineering Institute, Nanjing, China). 0.1 g of jejunal mucosa was weighed and added to 900 μL of homogenization medium (supplied with the kit). After centrifugation at 5,000 × *g* and 4 °C for 15 min, the supernatant was collected for subsequent use. Trypsin activity was measured using the ultraviolet colorimetric method, with an LOD of 3 U/mg prot, an intra-assay CV of ≤ 5.0% and an inter-assay CV of ≤ 8.0%. LPS activity was detected using the methylumbelliferone substrate method, with an LOD of 15 U/L (linear range: 15–300 U/L), an intra-assay CV of ≤ 6.0% and an inter-assay CV of ≤ 10.0%. Amylase was detected with an LOD of < 0.1 U/mL, an intra-assay CV of < 10%, and an inter-assay CV of < 15%. All procedures were performed in accordance with the manufacturer’s protocols. Enzyme activities were typically expressed as units per milligram of protein or per gram of tissue.

### Milk composition analysis

The milk samples were incubated in a water bath at 37 °C for 15 min, maintaining a constant temperature, and were thoroughly vortexed to ensure homogeneity. Subsequently, detection was carried out in strict accordance with the standard operating procedure of an automated milk analyzer (BOND, EKOMILK, Bulgaria). The contents of milk fat, milk protein, milk lactose and solids not fat (SNF) were determined, with all results being generated automatically by the instrument.

### Free amino acids in milk and plasma

1 mL of the sample was added to 3 mL of pre-cooled 10% sulfosalicylic acid solution. After vortex mixing, the mixture was kept at 4 °C for 5 min, after which it was centrifuged at 10,000 × *g* and 4 °C for 15 min. The resulting supernatant was filtered through a 0.45-µm membrane filter and analyzed using an automatic amino acid analyzer (L8900, Hitachi, Japan). It should be noted that milk samples required two filtration steps before analysis.

### Intestinal morphology examination

Intestinal tissue sections were prepared using methods previously described [20]. The tissue samples were fixed with 4% paraformaldehyde and processed using traditional paraffin embedding techniques. The tissue was then cut into 5-µm-thick sections and stained with hematoxylin and eosin. Villus height (VH) and crypt depth (CD) were visualized using an Eclipse E100 light microscope (Nikon, Tokyo, Japan) equipped with a DS-U3 digital imaging system (Nikon, Tokyo, Japan), and measured using CaseViewer software (3DHISTECH Ltd., Budapest, Hungary). Visual fields displaying intact villus morphology perpendicular to the intestinal wall and a clear crypt structure were selected. VH was defined as the vertical distance from the villus tip to the villus-crypt junction and CD as the vertical distance from the villus-crypt junction to the crypt base. The VH/CD ratio (VCR) was then calculated. Ten well-arranged and intact villi were randomly selected for measurement in each tissue section.

### Western blot analysis

A jejunal tissue sample of precisely 0.1 g was weighed, and 500 μL of pre-cooled RIPA lysis buffer containing 1% PMSF was added. The mixture was then placed immediately on an ice bath to prevent protein degradation. Homogenization was performed at 60 Hz for 60 s per cycle, repeated three to five times until the tissue was completely lysed. After homogenization, the suspension was centrifuged at 12,000 × *g* and 4 °C for 15 min. The collected supernatant was used as the total tissue protein extract and its concentration was determined using a BCA protein assay kit (ComWin Biotech, China). The protein samples were denatured and separated by SDS-PAGE before being transferred onto nitrocellulose membranes. The membranes were blocked with 5% skimmed milk powder at room temperature for 2 h, and then incubated with specific primary antibodies overnight at 4 °C. Information on the primary antibodies is provided in Table 2. Following incubation with the relevant HRP-conjugated secondary antibodies, the protein bands were visualized using an enhanced chemiluminescence (ECL) substrate (NCM Biotech, China) and imaged using an Amersham ImageQuant 800 system (Cytiva, Sweden). Band intensities were quantified using ImageJ software (National Institutes of Health, USA) and normalized to the corresponding β-actin levels.

**Table 2:** Antibodies used in this study

Target protein	Antibody type	Source	Catalog No.	Working concentration
ZO-1	Primary antibody	CST	#13663	1:1,000
p-mTOR	Primary antibody	CST	#4866	1:1,000
p-4EBP1	Primary antibody	CST	#2855	1:1,000
p-S6	Primary antibody	CST	#9234	1:1,000
Cytochrome C	Primary antibody	CST	#4272	1:1,000
NDUFS1	Primary antibody	CST	#70264	1:1,000
UQCRFS1	Primary antibody	CST	#95231	1:1,000
COX IV	Primary antibody	CST	#4850	1:1,000
COX10	Primary antibody	CST	#62387	1:1,000
OPA1	Primary antibody	CST	#80471	1:1,000
PHB1	Primary antibody	CST	#2426	1:1,000
SDHA	Primary antibody	CST	#5839	1:1,000
Occludin	Primary antibody	HuaBio	#R1510-33	1:1,000
Claudin-1	Primary antibody	HuaBio	#RT1141	1:1,000
SLC7A5/LAT1	Primary antibody	Santa	32683T	1:800
MFF	Primary antibody	CST	84580T	1:1,000
MFN2	Primary antibody	Santa	sc-515647	1:800
β-actin	Primary antibody	Beyotime	#AF5003	1:1,000
HRP conjugated Goat Anti-Rabbit IgG (H + L)	Secondary Antibody	Servicebio	GB23303	1:10,000
HRP conjugated Goat Anti-Mouse IgG (H + L)	Secondary Antibody	Servicebio	GB23301	1:10,000

### Milk metabolomic analysis

Fresh milk was collected under sterile conditions and stored on ice. It was then centrifuged at 12,000 × *g* for 10 min at 4 °C to remove the milk fat and any other precipitates. The resulting supernatant was divided into smaller portions and stored at −80 °C to avoid repeated freeze–thaw cycles. The frozen samples were thawed slowly on ice. 100 μL of the sample was mixed with 500 μL of pre-cooled methanol/water (4:1, v/v) extraction buffer, vortexed for 1 min, incubated at room temperature for 10 min, and stored at −20 °C overnight for protein precipitation. After centrifugation at 4,000 × *g* for 20 min at 4 °C, the supernatant was filtered through a 0.22-µm organic filter membrane. Metabolites were separated and detected using an UltiMate 3000 UPLC System (Thermo Fisher Scientific, Bremen, Germany) in conjunction with a TripleTOF 6600 high-resolution tandem mass spectrometer (SCIEX, Framingham, MA, USA). The raw data were processed using Progenesis QI software (Waters, USA; version 2.0) for peak alignment, normalization and compound identification. Metabolites were annotated by querying the METLIN, HMDB and Majorbio databases. Principal component analysis (PCA) was performed using the OmicStudio platform (https://www.omicstudio.cn/tool). Pathway enrichment analysis was conducted based on the KEGG database via MetaboAnalyst 6.0. Spearman's correlation analysis was performed to evaluate the relationships between differentially abundant gut microbiota, differential milk metabolites, average daily gain (ADG) and intestinal-related indices in suckling piglets.

### Microbiota profiling

As previously described [21], total DNA was isolated from the intestinal contents using commercial DNA extraction reagents (E.Z.N.A. and Soil DNA kits; Omega Bio-Tek, Norcross, GA, USA). DNA quality was assessed using electrophoresis on 1% agarose gels. Region-specific amplification targeting the 16S rRNA V4–V5 segment was then performed using custom-designed barcoded primers. The PCR products were subsequently detected using 2% agarose gels. The PCR products from the 2% agarose gel were then extracted and purified using the AxyPrep DNA Gel Extraction Method (Axygen Biosciences, Union City, CA, USA). Amplicon quantification was conducted using the QuantiFluor-ST Blue fluorometric system (Promega) and the amplicons were then mixed in proportion to the sequencing volume required for each sample. Sequencing libraries were generated from Illumina PE250 genomic DNA libraries according to standard protocols and sequenced on the Illumina PE250 platform (Shanghai BIOZERON Co., Ltd.). Raw sequencing reads underwent initial processing involving quality filtering and merging of paired-end sequences. Uparse software (Uparse v7.0.1001) was then used for OTU taxonomic analysis and cluster analysis, grouping together sequences with more than 97% similarity. The α and β diversity were analyzed based on the results of the OTU cluster analysis. The α diversity indices (Chao1, ACE, Shannon and Simpson) were computed using MOTHUR software (https://mothur.org/). Finally, we performed principal co-ordinates analysis (PCoA) and non-metric multi-dimensional scaling (NMSD) on the data using the Biozeron cloud platform for visualization.

### Statistical analysis

Data analyses were performed using SPSS 26.0 (IBM Corp., USA) and GraphPad Prism 9 (GraphPad Software, USA). One-way analysis of variance (ANOVA) was used to analyze comparisons across all dietary groups (Con, Arg, 20%Cit, 30%Cit and 40%Cit), followed by Duncan's multiple range test for post hoc comparisons. Prior to one-way ANOVA, normality was assessed using the Shapiro–Wilk test, with *P* > 0.05 indicating a normal distribution. For data with a normal distribution, homoscedasticity was verified using Levene's test (*P* > 0.05 indicating homogeneity of variance). If homoscedasticity was not met, Tamhane's T2 test was used for the subsequent statistical analysis instead of traditional ANOVA. Data that did not satisfy the normality assumption were analyzed using the Kruskal–Wallis test, followed by Bonferroni correction for post hoc multiple comparisons. Outliers were identified and excluded using the mean ± 3 standard deviations criterion. Spearman's correlation analysis was performed using the OmicStudio platform to assess the relationships between the variables. Differences were considered statistically significant at* P* < 0.05*,* with tendencies indicated by 0.05 ≤ *P* < 0.10,

## Results

### Maternal Cit supplementation enhances lactation performance by modulating milk composition

As shown in Table 3, the Arg and Cit groups had a markedly higher ADFI than the Con group (*P* < 0.01). The ADFI of the Arg, 20%Cit, 30%Cit and 40%Cit groups increased by 8.35%, 15.97%, 11.37% and 14.24%, respectively. On d 21 of lactation, the BW of sows in the 30%Cit and 40%Cit groups was significantly higher (*P* < 0.05) than that of the Arg group, increasing by 8.9% and 6.78%, respectively. Additionally, the 40%Cit group experienced lower BW loss than the Con group (*P* < 0.05). Consequently, suckling piglets’ ADG increased in response to maternal Arg and Cit supplementation (*P* = 0.086). No significant differences were observed among groups in terms of initial litter weight, piglet litter weight, weaning retention rate or number of weaned piglets. Therefore, dietary Cit supplementation during lactation may increase piglet ADG and improve sow lactation performance.

**Table 3:** Effects of maternal supplementation with Cit on sow performance during lactation^1^

Item	Treatments^2^					SEM	*P*-value
	Con	Arg	20%Cit	30%Cit	40%Cit		
Sow backfat, mm							
After farrowing	14.88	15.05	14.80	14.99	14.34	0.74	0.945
Day 21 lactation	14.25	14.67	14.46	14.56	14.43	0.32	0.995
Backfat loss	0.63	0.49	0.34	0.44	−0.10	0.21	0.850
Sow BW, kg							
Starting	245.81	236.88	249.36	252.27	241.31	2.62	0.342
Day 21 lactation	239.38^bc^	235.00^c^	245.00^abc^	255.93^a^	250.93^ab^	2.25	0.020
BW loss	6.44^a^	1.88^ab^	4.36^ab^	−3.65^ab^	−9.61^b^	1.83	0.036
Starting							
Initial piglet weight, kg	1.52	1.56	1.52	1.53	1.55	0.05	0.983
Initial litter weight, kg	18.27	18.71	18.25	18.31	18.58	0.64	0.984
Initial number of piglets, n	12	12	12	12	12		
Day 21 lactation							
Piglet litter weight, kg	63.09	68.51	68.94	68.04	69.24	2.16	0.292
Number of weaned piglets, n	11.54	11.85	11.59	11.67	11.63	0.15	0.628
ADG, g/d	217.99	235.11	246.87	237.63	245.44	8.04	0.086
Weaning Retention Rate, %	94.89	97.93	95.5	96.99	96.4	1.43	0.548
Sow ADFI, kg/d							
Days 3–21 lactation	6.95^c^	7.53^b^	8.06^a^	7.74^ab^	7.94^ab^	0.15	< 0.001

*SEM* Standard error of the mean, *ADFI* Average daily feed intake, *ADG* Average daily weight gain, *BW* Body weight, *Starting* All sows had finished farrowing

^1^Mean and total SEM are list in separate columns, *n* = 30

^2^*Con* Control, *Arg* Diet with arginine, *20%Cit* Diet with 20% Arg replaced by L-Citrulline, *30%Cit* Diet with 30% Arg replaced by L-Citrulline, *40%Cit* Diet with 40% Arg replaced by L-Citrulline

^a–c ^Means within a row with different superscripts differ significantly (*P* < 0.05, Duncan’s test)

To explore the effects of maternal Cit supplementation on the endocrine homeostasis and physiological status of lactating sows, we examined certain systemic physiological parameters. Compared with the Arg group, maternal supplementation with 40%Cit significantly decreased plasma progesterone content on d 21 of lactation (*P* < 0.05). There were no significant changes in prolactin, insulin or estradiol levels (Table 4). Furthermore, the Arg group and the 40%Cit group demonstrated a substantial increase in plasma NO levels (*P* < 0.05) compared with the Con group, the 20%Cit group and the 30%Cit group. This indicates enhanced metabolic and vascular function. As shown in Table 5, plasma Cit concentration in sows was significantly higher in the 40%Cit group than in the Con and Arg groups (*P* < 0.05). Similarly, plasma Arg concentration in sows was significantly higher in the 30%Cit group than in the Con and 20%Cit groups (*P* < 0.05). The Glu content showed an upward trend (*P* = 0.082). However, there were no differences in the plasma levels of Lys, Thr, Glu, Gln, Ile and Leu among the groups. These changes in maternal physiological status were reflected in milk composition. Compared with the Con group, milk fat content significantly increased in both the Arg group and the 40%Cit group (*P* < 0.05; Table 6), while the composition of other milk components remained unaffected. In addition, the content of free amino acids in milk was determined (Table 5). The results showed that Arg content remained unchanged, Thr content tended to increase (*P* = 0.084), and Leu content tended to decrease (*P* = 0.089). However, there were no differences in the levels of Tau, Glu, Gln and Val in the milk among the groups.

**Table 4:** Effects of maternal supplementation with Cit on plasma hormones of sows (*n* = 8)

Item	Treatments^1^					SEM	*P*-value
	Con	Arg	20%Cit	30%Cit	40%Cit		
Estradiol, pmol/L	92.82	81.81	91.37	86.16	92.71	4.71	0.943
Progesterone, pmol/L	2,009.02^ab^	2,088.71^a^	2,275.14^ab^	2,106.01^ab^	1,657.12^b^	78.53	0.020
Prolactin, ng/mL	9.21	6.39	9.31	7.82	8.65	0.51	0.365
Insulin, mIU/L	83.29	89.57	88.86	79.06	81.10	1.78	0.237
NO, μmol/L	0.73^b^	1.60^a^	0.61^b^	0.60^b^	1.42^a^	0.12	0.003

*NO* Nitric oxide

^1^*Con* Control, *Arg* Diet with arginine, *20%Cit* Diet with 20% Arg replaced by citrulline, *30%Cit* Diet with 30% Arg replaced by citrulline, *40%Cit* Diet with 40% Arg replaced by citrulline

^a,b ^Means with different superscripts within a row differ significantly (*P* < 0.05, Kruskal–Wallis test with Bonferroni correction)

**Table 5:** Effects of maternal Cit supplementation on milk and plasma free amino acid concentrations in lactating sows (*n* = 8)

Item, μmol/L	Treatments^1^					SEM	*P*-value
	Con	Arg	20%Cit	30%Cit	40%Cit		
Plasma							
Cit	80.00^c^	95.43^bc^	99.43^abc^	114.86^abc^	154.29^a^	5.76	0.001
Arg	93.33^c^	133.71^ab^	104.00^bc^	160.67^a^	116.86^abc^	6.14	0.004
Lys	119.33	125.43	118.86	152.00	136.00	5.78	0.332
Thr	122.00	130.29	103.67	129.14	121.14	5.40	0.557
Glu	167.71	188.57	181.60	206.29	238.29	8.77	0.082
Gln	322.86	270.86	360.57	360.00	314.86	13.48	0.192
Ile	95.43	94.67	96.00	91.43	96.00	1.93	0.949
Leu	144.00	168.00	181.14	194.29	158.86	6.95	0.176
Milk							
Arg	155.00	164.00	149.75	171.55	162.50	7.85	0.929
Thr	205.00	390.00	245.00	400.00	345.00	30.97	0.084
Leu	55.50	46.00	35.00	52.50	40.00	2.72	0.089
Tau	2,328.00	2,539.00	3,117.00	2,705.00	2,600.50	116.90	0.307
Glu	467.00	572.00	450.00	415.50	540.00	27.93	0.375
Gln	108.50	119.00	141.00	134.50	148.25	8.17	0.550
Val	53.00	59.00	46.25	53.50	59.50	2.13	0.258

^1^*Con* Control, *Arg* Diet with arginine, *20%Cit* Diet with 20% Arg replaced by citrulline, *30%Cit* Diet with 30% Arg replaced by citrulline, *40%Cit* Diet with 40% Arg replaced by citrulline

^a–^^c^ Means with different superscripts within a row differ significantly (*P* < 0.05, Kruskal–Wallis test with Bonferroni correction)

**Table 6:** Effects of maternal supplementation with Cit on milk composition of sows (*n* = 10)

Item	Treatments^1^					SEM	*P*-value
	Con	Arg	20%Cit	30%Cit	40%Cit		
Milk protein, %	4.15	4.14	4.26	4.22	4.29	0.04	0.808
Milk fat, %	4.91^b^	6.07^a^	6.47^a^	6.89^a^	6.42^a^	0.18	0.003
Lactose, %	5.79	5.95	6.11	6.05	6.16	0.07	0.441
SNF, %	10.62	10.94	11.23	11.10	11.32	0.13	0.446

*SNF* Non-fat solids

^1^*Con* Control, *Arg* Diet with arginine, *20%Cit* Diet with 20% Arg replaced by citrulline, *30%Cit* Diet with 30% Arg replaced by citrulline, *40%Cit* Diet with 40% Arg replaced by citrulline

^a,b ^Means within a row with different superscripts differ significantly (*P* < 0.05, Duncan's test)

### Maternal 40%Cit supplementation improves intestinal health in suckling piglets

**Fig. 1:**
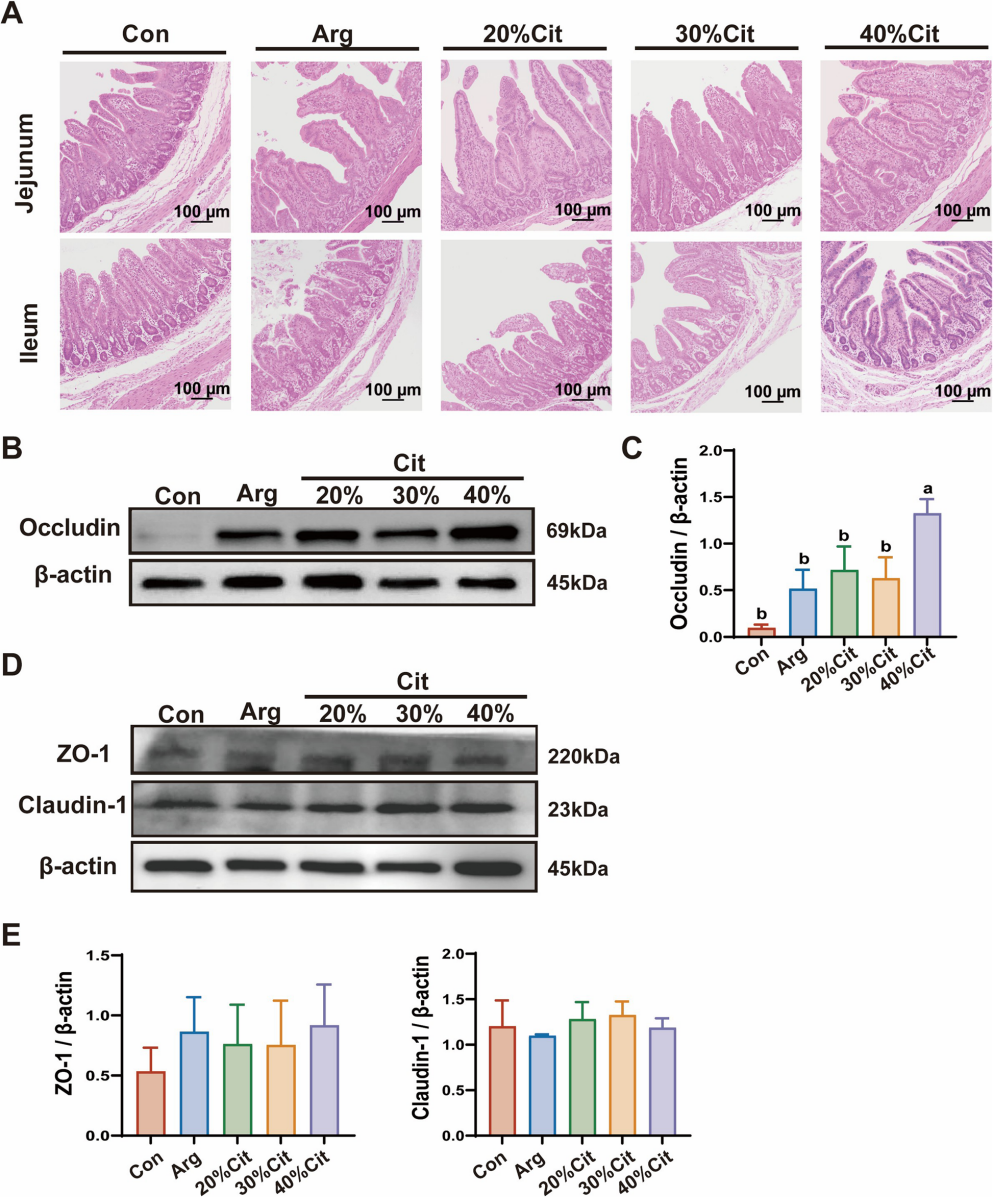
Maternal Cit supplementation enhances the intestinal health of suckling piglets during lactation. **A** Representative hematoxylin–eosin (H&E)-stained images of the jejunum and ileum (magnification: 20 ×). **B**–**E** Protein expression levels of jejunal tight junction proteins (*n* = 3). Data are presented as mean ± standard error of the mean (SEM). Different letters (a, b) above the data indicate significant differences (*P* < 0.05)

Based on the observed improvements in milk composition and growth trends in piglets, we hypothesized that maternal Cit supplementation enhances offspring health by supporting intestinal development. Therefore, we first examined the morphology of the jejunum and ileum of suckling piglets. As shown in Fig. 1A and Table 7, the 40%Cit group exhibited clear improvements in jejunal architecture. VH, CD and VCR are important indicators for evaluating intestinal structure. In general, a higher VH and VCR, together with a lower CD, indicate better intestinal function [24]. As shown in Table 7, the Arg, 20%Cit and 40%Cit groups showed a significant increase in jejunal VH compared with the Con group (*P* < 0.05). Compared with the 30%Cit group, the 40%Cit group reduced jejunal CD (*P* < 0.05). Jejunal VCR increased significantly in the 40%Cit group compared with the Con and 30%Cit groups (*P* < 0.05).

**Table 7:** Effects of maternal Cit supplementation on intestinal villus morphology of piglets (*n* = 10)

Item	Treatments^1^					SEM	*P*-value
	Con	Arg	20%Cit	30%Cit	40%Cit		
Ileum							
VH, mm	378.66	431.40	358.45	429.28	446.51	16.69	0.217
CD, mm	139.92	149.28	130.68	168.85	153.63	5.70	0.160
VCR	2.75	3.07	2.83	2.56	2.91	0.10	0.615
Jejunum							
VH, mm	359.65^c^	592.45^a^	534.60^ab^	413.48^bc^	527.57^ab^	25.87	0.020
CD, mm	162.11^ab^	169.17^ab^	175.80^ab^	224.49^a^	116.53^b^	10.39	0.020
VCR	2.69^b^	3.64^ab^	3.97^ab^	2.02^b^	4.77^a^	0.26	0.004

*VH* Villus height, *CD* Crypt depth, *VCR* Villus height to crypt depth ratio

^1^*Con* Control, *Arg* Diet with arginine, *20%Cit* Diet with 20% Arg replaced by citrulline, *30%Cit* Diet with 30% Arg replaced by citrulline, *40%Cit* Diet with 40% Arg replaced by citrulline

^a–^^c^ Means within a row with different superscripts differ significantly (*P* < 0.05, Duncan's test). Kruskal–Wallis test was used for VCR of jejunum and CD of Ileum

We then investigated the underlying molecular and functional correlates of these morphological changes. Analysis of intestinal barrier function revealed that maternal Cit supplementation significantly increased occludin protein levels (*P* < 0.05), while the levels of ZO-1 and claudin-1 levels remained unchanged in the jejunum of suckling piglets (Fig. 1B–E). Additionally, trypsin activity exhibited an upward trend in the 40%Cit groups (*P* = 0.087; Table 8), though no significant effects were observed in AMS or LPS activities. In summary, maternal Cit supplementation, particularly at 40%, improved the intestinal health of sucking piglets by enhancing jejunal morphology, strengthening the intestinal barrier and increasing trypsin enzyme activity.

**Table 8:** Effects of maternal Cit supplementation on jejunal digestive enzyme activity in piglets (*n* = 6)

Item	Treatments^1^					SEM	*P*-value
	Con	Arg	20%Cit	30%Cit	40%Cit		
AMS, U/mgprot	2.88	2.78	2.76	3.58	2.22	0.19	0.335
LPS, U/gprot	39.10	37.58	30.18	35.87	36.70	1.90	0.552
Trypsin, U/mgprot	5.35	6.27	6.32	6.36	8.78	0.44	0.087

*AMS* Amylase, *LPS* Lipase

^1^*Con* Control, *Arg* Diet with arginine, *20%Cit* Diet with 20% Arg replaced by citrulline, *30%Cit* Diet with 30% Arg replaced by citrulline, *40%Cit* Diet with 40% Arg replaced by citrulline

A tendency was declared at 0.05 ≤ *P* < 0.10. Kruskal–Wallis test was used for AMS and LPS, Duncan’s test was used for trypsin

### Maternal 40%Cit supplementation improves the intestinal health of offspring via the mTOR/S6 pathway, as well as the structural and functional status of mitochondria

**Fig. 2:**
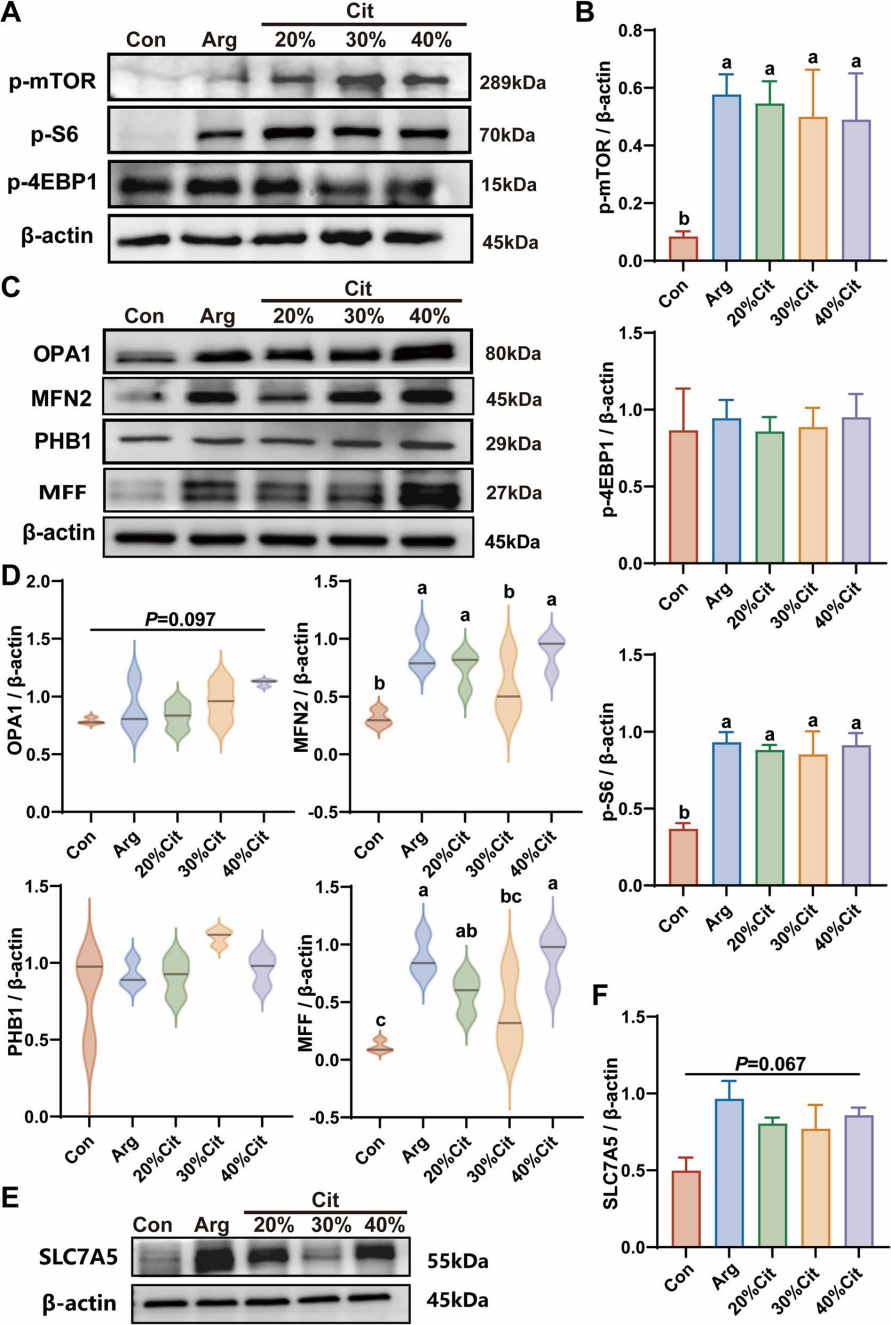
Effects of maternal Cit supplementation on phosphorylated mTOR proteins and mitochondrial functional proteins in the jejunal tissue of suckling piglets. **A** and **B** The expression levels of phosphorylated proteins in the mTOR signaling pathway within the jejunal intestinal tissue of suckling piglets. **C** and **D** Expression levels of structural and functional mitochondrial proteins. **E** The expression of Cit transporter. All data are presented as mean ± SEM (*n* = 3). Different letters (a, b, c) above the data indicate significant differences (*P* < 0.05). A tendency toward significance is indicated when 0.05 ≤ *P* < 0.1. *p-mTOR* Phosphorylated-mammalian target of rapamycin, *p-S6* Phosphorylated-ribosomal protein S6 kinase, *p-4EBP1* Phosphorylated-4E-binding protein 1, *OPA1* Optic atrophy 1, *MFN2* Mitofusin-2, *PHB1* Prohibitin 1, *MFF* Mitochondrial fission factor, *SLC7A5* Solute carrier family 7 member 5

To investigate the mechanisms underlying the improved intestinal health in suckling piglets, we examined the mTOR signaling pathway and mitochondrial function. As shown in Fig. 2A and B, the phosphorylation of mTOR and S6 proteins in the jejunum of suckling piglets were significantly higher in the Arg, 20%Cit, 30%Cit and 40%Cit groups than in the Con group, while the phosphorylation level of 4EBP1 remained unchanged. Furthermore, Fig. 2C shows that the 40%Cit group exhibited a tendency towards increased OPA1 expression (*P* = 0.097), which is a key regulator of mitochondrial fusion and structural integrity. Compared with the Con group, the expression of MFN2 and MFF increased significantly in the Arg, 20%Cit and 40%Cit groups (*P* < 0.05), but the expression of PHB1 was not significantly altered (Fig. 2C and D). Additionally, no significant differences were observed in the expression of proteins related to mitochondrial metabolic function (COXIV, COX10, Cytochrome c, NDUFS1 and UQCRFS1; Fig. S1). The protein expression level of SLC7A5, a Cit transport carrier in the jejunum of suckling piglets, showed an increasing trend in the 40%Cit group (*P* = 0.067, Fig. 2E and F). These results suggest that maternal Cit supplementation activates the mTOR/S6 signaling pathway and improves mitochondrial dynamic balance, thereby enhancing its structural and functional status. Based on these pronounced effects, the 40%Cit group was selected for further mechanistic investigation.

### Maternal 40%Cit supplementation modulates gut microbiota in suckling piglets

**Fig. 3:**
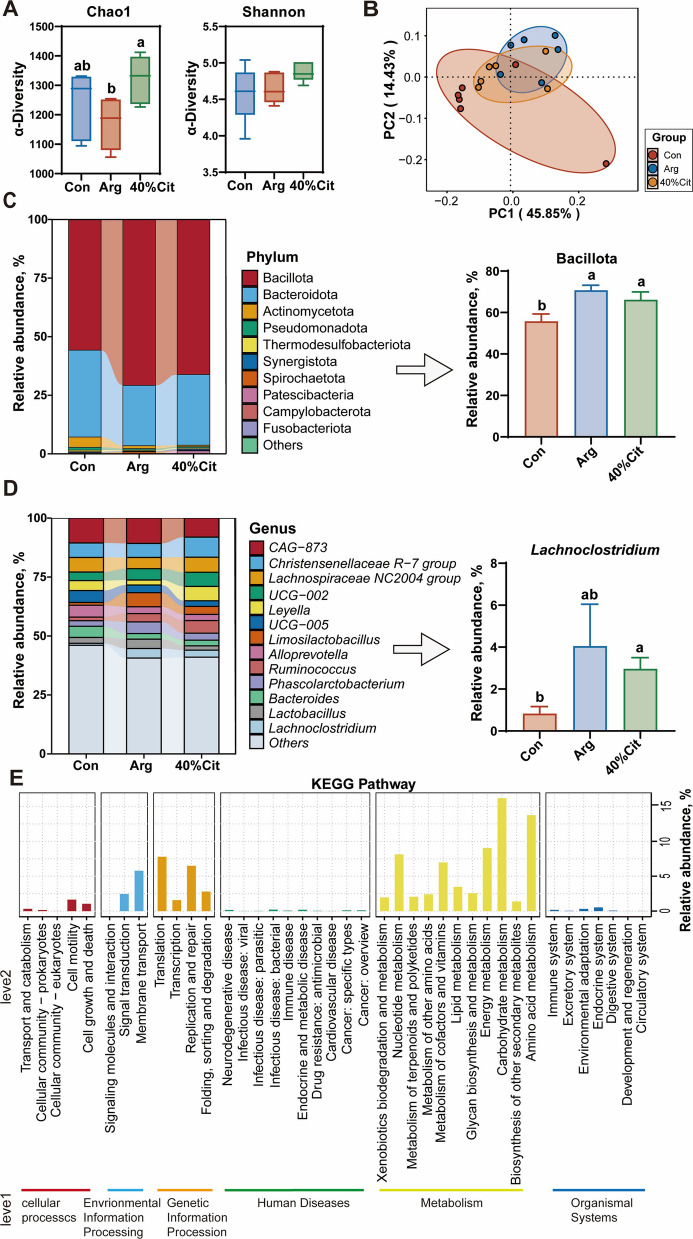
Maternal 40%Cit supplementation modulates gut microbiota composition in suckling piglets during lactation. **A** Chao1 and Shannon indices of gut microbiota diversity. **B** PCoA plot of gut microbiota based on Bray–Curtis distances. **C** and **D** Relative abundance of microbial communities at phylum and genus levels. **E** Bar chart showing the distribution of KEGG pathway annotations at Level 1 and Level 2. Based on the functional prediction results from PICRUSt2, the relative abundance of KEGG pathways in each sample or subgroup was calculated at Level 1 and Level 2, respectively. Different letters (a, b) above the data indicate significant differences (*P* < 0.05, *n* = 6)

Given the importance of intestinal microbial balance for gut health, we investigated the impact of maternal supplementation with Arg and 40%Cit on the gut microbiota of suckling piglets. As shown in Fig. 3A, the Chao1 index significantly increased in the 40%Cit group compared with the Arg group (*P* < 0.05), while the Shannon index showed no significant difference. PCoA revealed minimal separation among the three groups (Fig. 3B). At the phylum level, the relative abundance of Bacillota increased significantly in both the Arg and 40%Cit groups (*P* < 0.05; Fig. 3C). Significant alterations were observed at genus level: the relative abundance of *Lachnoclostridium* in 40%Cit group increased significantly (*P* < 0.05), compared with the Con group (Fig. 3D). In addition, there was no significant change in the relative abundance of *Lachnoclostridium* compared with the Arg group. Functional prediction using PICRUSt2 identified 6 primary pathways and 40 secondary pathways (Fig. 3E). This suggests remodeling of the microbial functional profile, particularly with regard to carbohydrate and amino acid metabolism.

**Fig. 4:**
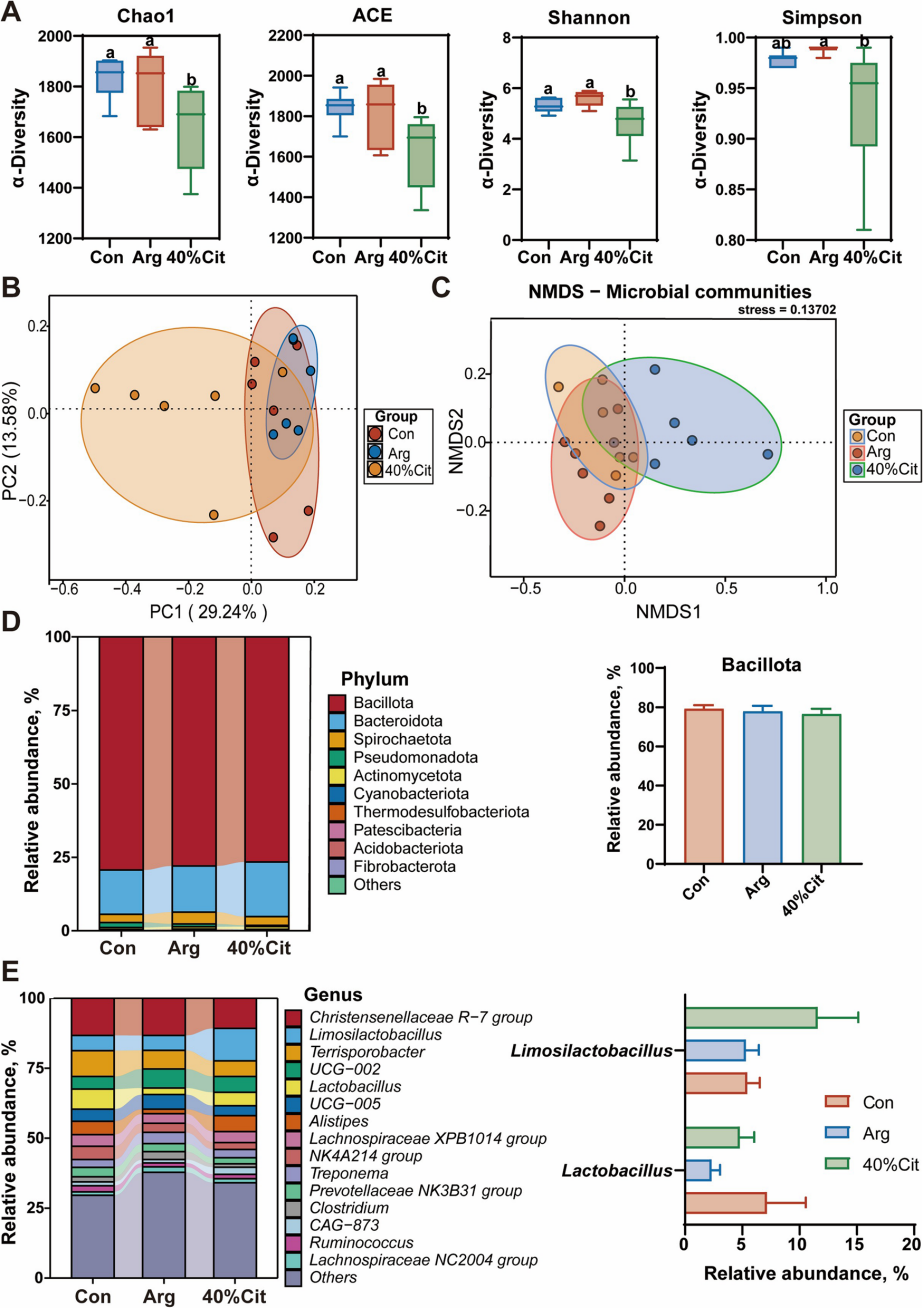
Maternal 40%Cit supplementation impacts sow microbiota diversity and suckling piglet gut microbiota function prediction. **A** α diversity results. **B** PCoA plot displaying β diversity among microbiota samples. **C** NMDS showing β diversity among microbiota samples. **D** Relative abundances of main taxa at phylum. **E** Relative abundances of main taxa at genus. Different letters (a, b) above peer data indicate significant differences (*P* < 0.05, *n* = 6)

Maternal supplementation with 40%Cit significantly reduced microbial richness in sows, as shown by decreased Chao1, ACE and Shannon indices compared with the Con and Arg groups (*P* < 0.05; Fig. 4A). Meanwhile, the Simpson index in the 40%Cit group was significantly decreased, compared with the Arg group. However, PCoA and NMDS analyses showed no significant differences in β-diversity among the groups (Fig. 4B and C), and the composition of the dominant microbiota at phylum and genus levels were no significant changes (Figs. 4D and E). Furthermore, the significant differences in microbial communities observed in the piglets’ intestines were not accompanied by corresponding changes in the sows’ feces, suggesting that the alterations in the piglets’ gut microbiota were not due to direct transmission of microorganisms from the sows’ feces. The results of milk microbial detection showed that there was no significant change in the relative abundance of microbiota at different levels of sows supplemented with Arg and 40%Cit maternally (Fig. S2). Taken together, these results demonstrate that maternal supplementation with 40%Cit during lactation specifically modulates the composition of the gut microbiota and metabolic functions in suckling piglets while having limited effects on the maternal microbial community.

### Maternal 40%Cit supplementation alters the milk metabolome

**Fig. 5:**
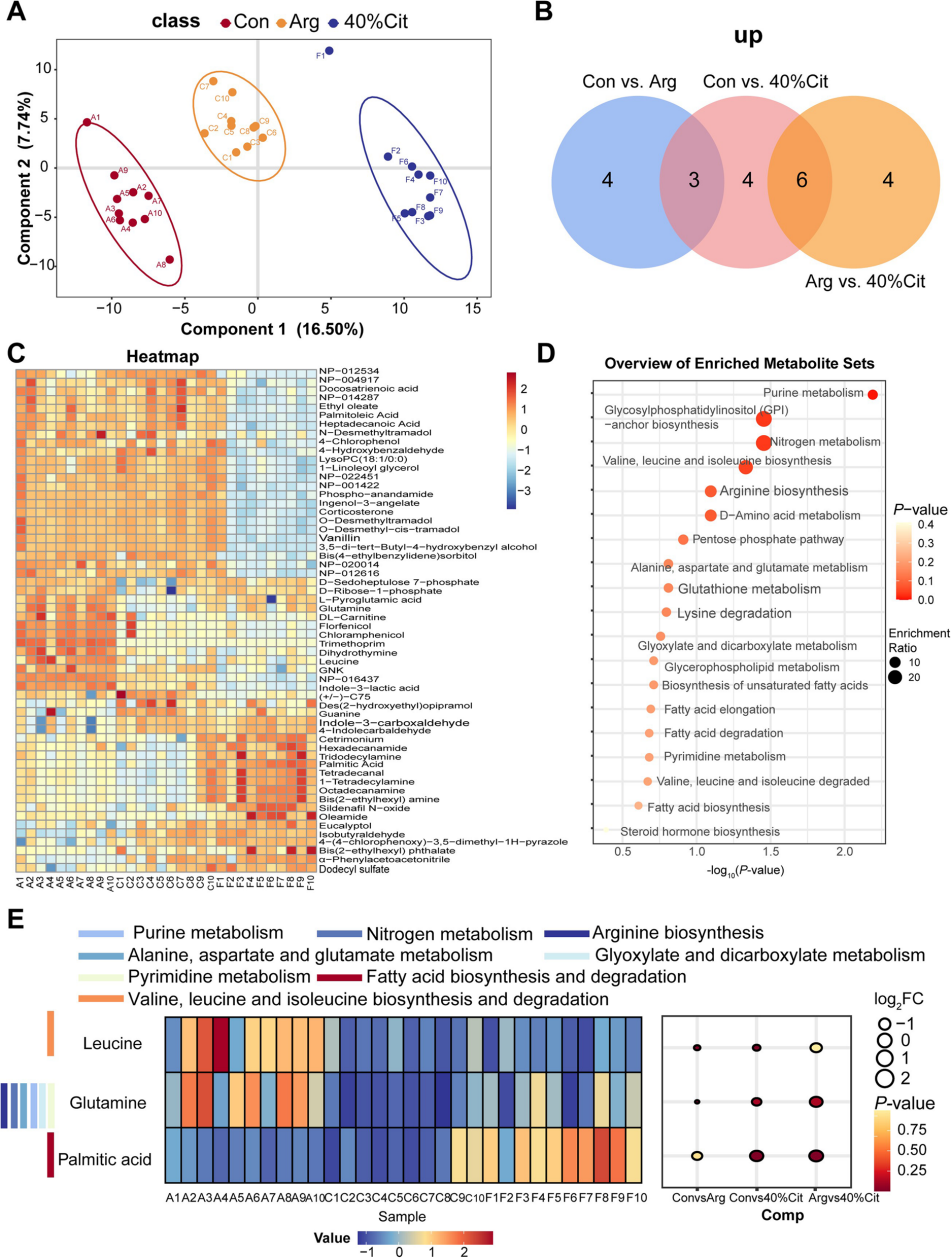
Effects of maternal 40%Cit on the metabolites in milk. **A** PLS-DA analysis. **B** The Venn diagram shows the significantly upregulated differential metabolites (Con vs. Arg, Con vs. 40%Cit and Arg vs. 40%Cit). **C** Heatmap of differential metabolites. **D** Bubble plot of KEGG pathway enrichment. **E** Advanced heatmap barplot of differential metabolites with KEGG annotations (*n* = 10)

Milk is the primary nutritional source for newborn piglets, containing various bioactive metabolites that support intestinal health. Untargeted metabolomic analysis revealed that maternal supplementation with 40%Cit significantly altered the milk metabolic profile. Partial least squares discrimination analysis (PLS-DA) showed that samples from the Con group, Arg group and 40%Cit group showed a clear tendency to separate in the principal component space. The model fit parameters (R^2^ = 0.99 and Q^2^ = 0.90) indicating that the model possesses good explanatory and predictive capabilities. Validated by a 200-replication bootstrap test, the intercept of the Q^2^ regression line on the *y*-axis is negative (−0.62), confirming that the model does not exhibit overfitting. These results indicate that there are significant differences in metabolic profiles among the groups (Fig. 5A). We compared the three groups in pairs to identify differentially expressed metabolites (fold change > 2.0, *P* < 0.05; Fig. S3). We focused primarily on upregulated metabolites, of which there were 30 in total (Fig. 5B). As shown in Fig. 5C and D, the former illustrates changes in the content of differential metabolites in milk, and the latter shows the results of pathway enrichment analysis. Three differential metabolites (DMs) were annotated in the Kyoto Encyclopedia of Genes and Genomes (KEGG) database in total. Notably, the content of palmitic acid, a key metabolite in fatty acid biosynthesis and degradation, increased significantly in the 40%Cit group. Conversely, the levels of leucine and glutamine decreased markedly (Fig. 5E).

### Correlation analysis of intestinal differential microbiota of suckling piglets and ADG, milk metabolites and composition, intestinal barrier indicators, the mTOR signaling pathway and mitochondrial structural and functional protein

**Fig. 6:**
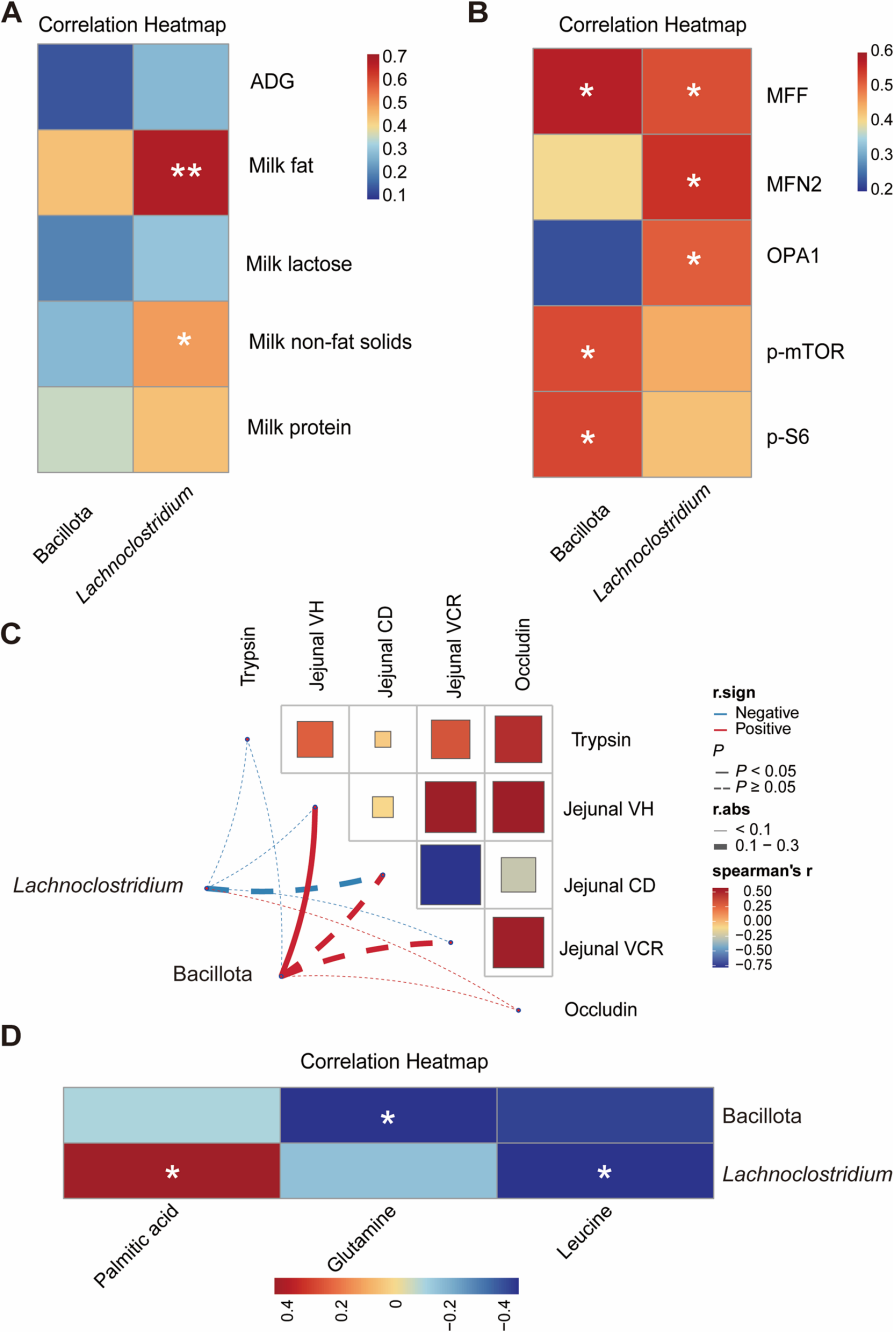
Correlation analysis of the intestinal differential microbiota of suckling piglets with ADG, milk composition, intestinal barrier indicators, the mTOR signaling pathway, mitochondrial structural and functional proteins. **A** Spearman’s correlation analysis of milk components and ADG with differential microbial communities. **B** Spearman's correlation analysis between the intestinal microbiota and the intestinal barrier indices, the mTOR signaling pathway, and the mitochondrial structural and functional proteins. **C** Spearman's correlation analysis between the intestinal microbiota of suckling piglets and their intestinal indices. **D** Spearman's correlation analysis between the differential intestinal microbiota of suckling piglets and the differential metabolites in milk. In the heatmap of the correlation coefficient, red represents positive correlations and blue represents negative correlations. ^*^P < 0.05, ^**^P < 0.01

Correlation analysis was performed to elucidate the relationships between intestinal differential microbiota at the phylum and genus levels, ADG and milk composition. Significant positive correlations were observed between milk fat (*P* < 0.01) and SNF (*P* < 0.05) contents and the relative abundance of *Lachnoclostridium* (Fig. 6A). Regarding intestinal morphology and barrier function (Fig. 6B and C), we found the expression levels of MFF, p-mTOR, and p-S6 proteins were significantly positively correlated with the relative abundance of Bacillota (*P* < 0.05); the expression levels of MFF, MFN2, and OPA1 proteins were significantly positively correlated with the relative abundance of *Lachnoclostridium* (*P* < 0.05; Fig. 6B). The jejunal VH is significantly positively correlated with the relative abundance of Bacillota (*P* < 0.05; Fig. 6C).

Correlation analysis revealed significant relationships between these milk metabolites and intestinal microbiota in suckling piglets. Glutamine showed significant negative correlation with Bacillota (*P* < 0.05), and leucine displayed a significant negative correlation with *Lachnoclostridium* (*P* < 0.05). Conversely, palmitic acid demonstrated a significant positive correlation with *Lachnoclostridium* (*P* < 0.05; Fig. 6D).

## Discussion

Cit, an effective Arg precursor, bypasses hepatic catabolism and is almost entirely absorbed into the systemic circulation. This enables efficient conversion to Arg in the kidneys, enhancing systemic bioavailability. Previous studies have shown that supplementing the diet of pregnant sows with Arg can significantly improve the growth performance of piglets, and that feed intake increases as Arg levels in the diet rise during lactation [3, 20, 22, 23]. Consistent with this, our study found that maternal supplementation of Cit, a precursor of Arg, improved ADFI of sows, decreased BW loss and tended to increase ADG of suckling piglets. Appropriate levels of Arg have been shown to promote appetite and feed intake in animals [24, 25]. It is worth noting that compared with supplementation of 20% or 30% Cit, 40% Cit significantly elevated plasma NO concentrations in sows, indicating a dose-dependent effect of Cit on NO production. Consistent with this observation, Schwedhelm et al. [26] demonstrated that oral Cit supplementation increases plasma Arg levels in a dose-dependent manner and potentiates NO-dependent signaling pathways. Cit is metabolized to arginine via the sequential enzymatic actions of argininosuccinate synthase and argininosuccinate lyase; Arg, in turn, serves as the immediate precursor for NO synthesis catalyzed by nitric oxide synthase. Notably, maternal plasma Arg concentration peaked at the 30% Cit supplementation level—likely reflecting a balance between enhanced Arg synthesis from increased Cit availability and accelerated Arg utilization as a substrate for NO generation. As Bahadoran et al. [27] reported, Cit exhibits superior bioavailability as an NO precursor under conditions of heightened metabolic demand.

Preliminary research has found that adding Cit can effectively reduce the pre-weaning mortality rate [13]. Our study further confirms that maternal supplementation with Cit improves milk quality, promotes colonization of beneficial gut microbiota in suckling piglets, and enhances their ADG and intestinal health. Cit is even more effective than direct Arg oral supplementation in increasing blood Arg concentration [26, 28]. Supplementing sows’ diets with Arg significantly enhances intestinal development in newborn piglets, as evidenced by a marked increase in VH and a significant reduction in CD [29]. We found that maternal supplementation with 40%Cit improved the morphological characteristics of the intestine and the expression of tight junction proteins in suckling piglets, resulting in increased VH and elevated VCR. This indicates that the effects of supplementing 40%Cit to the maternal diet and supplementing Arg to the maternal diet on the intestinal morphology of suckling piglets are similar. Previous studies have shown that Arg can increase milk fat content through NO-mediated blood flow, while also regulating the expression of genes related to mammary metabolism and promoting mammary development. Furthermore, it has been found to upregulate key genes involved in milk fat synthesis [22, 30, 31]. Similarly, we found that the content of NO and milk fat significantly increased in the Arg group, with an increase also observed in the 40%Cit group. Furthermore, supplementing Arg in the diet affects the levels of metabolites and free amino acids in milk [3, 32], which is consistent with our findings. In the present study, we also demonstrated that maternal supplementation with 40%Cit resulted in an augmentation of fatty acid compounds in milk, with the elevated palmitic acid primarily accumulating in the fatty acid biosynthesis pathway—the pivotal pathway for milk fat synthesis. Palmitic acid, a long-chain saturated fatty acid, is considered an ideal substitute for fatty acids in human milk due to its favorable taste and low sensitization potential [33]. Studies have shown that administering palmitic acid via milk gavage can improve metabolic function and reduce inflammation in rats [34]. Moreover, when esterified at the sn-2 position in milk, palmitic acid is rapidly absorbed by the intestine, thus encouraging energy and calcium uptake in offspring [35]. Gao [32] found that supplementing lactating sows' diets with Arg increased the levels of Arg and ornithine in milk, while significantly reducing Cit content. However, our study found that maternal Arg supplementation did not significantly alter Arg levels in milk, which is consistent with the findings of Mendes et al. [22]. Similarly, no Cit was detected in the milk of Cit groups. This may be because Cit, an endogenous Arg precursor, is primarily converted to Arg in the kidneys rather than in mammary tissue [36]. Moreover, dietary supplementation with amino acids has been demonstrated to assist in the regulation of the gut microbiota [2]. However, our study found that maternal supplementation of Arg or 40% Cit had no significant effect on the overall microbial composition of sow feces and milk. Nevertheless, the intestinal microbial abundance of *Lachnoclostridium* at the phylum level of Firmicutes significantly increased in piglets suckling milk from sows supplemented with 40%Cit. This finding is consistent with the results reported by Diana et al. [37], which suggest that amino acid supplementation during lactation can indirectly impact the gut microbiota of piglets. Furthermore, research has demonstrated that fatty acids regulate the colonization and development of gut microbiota [38, 39] and that the palmitic acid in milk can promote the growth of *Lactobacillus* [35, 40]. We found that the relative abundance of *Lachnoclostridium* is significantly positively correlated with the palmitic acid content in milk. This suggests that maternal transmission may promote the colonization of *Lachnoclostridium*. However, further investigation is required into the mechanisms underlying the increased palmitic acid in milk and gut microbes in suckling piglets following maternal supplementation with 40%Cit.

Amino acids can promote protein synthesis by activating the mTOR pathway [41]. Interestingly, our study found that the overall content of free amino acids in milk supplemented with maternal Cit did not change; only Leu showed a decreasing trend, while Thr content increased. This suggests that activation of the jejunal mTOR signaling pathway in piglets by maternal Cit supplementation may not be driven directly by milk amino acid levels, but by other mediating mechanisms. Taherian-Esfahani et al. [42] found that *Lactobacillus* strains can regulate the expression of proteins related to the mTOR and Wnt/β-catenin pathways in colon cancer cell lines, and a study by Ghanavati et al. [43] showed similar results. In our study, we found that the relative abundance of Bacillota in suckling piglets from sows supplemented with 40%Cit was significantly and positively correlated with the protein contents of p-mTOR and p-S6. This suggests that Bacillota may regulate the mTOR/S6 pathway through their metabolites. However, it remains to be ascertained whether bacteria in Bacillota can affect mTOR pathway protein expression in piglet intestinal cells under normal physiological conditions. Furthermore, mTOR has been shown to regulate mitochondrial function [44, 45]. Morita et al. [46] confirmed that mTORC1 activation selectively promotes the translation of nuclear-encoded, mitochondrial-related mRNA by inhibiting eIF4E-binding proteins, which play a key role in regulating mitochondrial activity and biogenesis. Mitochondrial fusion is mediated by MFN1, MFN2 and OPA1 [47, 48]. Fission is primarily driven by Drp1, which is recruited and activated by the outer membrane protein MFF. MFF binds directly to Drp1, promoting its assembly at fission sites [49]. In our study, maternal supplementation with 40%Cit activated the mTOR/S6 pathway in the jejunal tissue of suckling piglets, and tended to upregulate OPA1 expression. OPA1 is a key protein that maintains the structural and functional integrity of mitochondria [50]. Furthermore, increased protein levels of both MFN2 and MFF suggest that 40%Cit activates the mTOR/S6 pathway in the jejunum of suckling piglets, thereby improving the structural and functional homeostasis of mitochondrial fusion and fission to some extent. However, the mechanism by which mTOR activation regulates mitochondrial fusion and fission requires further investigation. It is noteworthy that recent studies have demonstrated a positive correlation between MFN2 expression levels and both intestinal barrier integrity and mitochondrial homeostasis. Upregulation of MFN2 expression levels represents a key protective mechanism for maintaining intestinal health [51].

## Conclusion

In conclusion, dietary Cit supplementation may be a viable strategy to improve sow lactation performance, as evidenced by enhanced milk fat and beneficial shifts in milk metabolite profiles (e.g., palmitic acid). These maternal improvements contribute to increased piglet ADG and intestinal health, likely through the upregulation of tight junction proteins (e.g., occludin), activation of the jejunal mTOR/S6 pathway, and improved mitochondrial structure and function in the piglet jejunum. Overall, our study reveals a potential mechanism whereby this effect is closely linked to regulating intestinal health in piglets, providing crucial theoretical support for the application of Cit as a feed additive in lactating sows.

## Supplementary Information

Additional file 1: Table S1. Selection indicators of sampled sows. Table S2. Selection indicators of sampled suckling piglets.

Additional file 2: Fig. S1. Effects of maternal Cit supplementation on the expression of proteins related to mitochondrial metabolic function. Fig. S2. Effects of maternal Cit supplementation on the microorganisms in milk. Fig. S3. Effects of maternal 40%Cit on the metabolites in milk.

Additional file 3. Original images for Western blot.

## Data Availability

The data used and analysed during the current study are available from the corresponding author on reasonable request.

## Abbreviations

ADFI: Average daily feed intake
ADG: Average daily gain
AMS: Amylase
Arg: Arginine
BW: Body weight
Cit: Citrulline
CD: Crypt depth
COX10: Cytochrome c oxidase assembly factor heme a: farnesyltransferase cox10
COX IV: Cytochrome c oxidase subunit 4
ILe: Isoleucine
Leu: Leucine
LPS: Lipase
mTOR: Mammalian target of rapamycin
MFF: Mitochondrial fission factor
MFN2: Mitofusin-2
NDUFS1: Ubiquinone oxidoreductase core subunit S1
NO: Nitric oxide
OPA1: Optic atrophy 1
P-S6: Phosphorylated-ribosomal protein S6 kinase
P-4EBP1: Phosphorylated-4E-binding protein 1
P-mTOR: Phosphorylated-mammalian target of rapamycin
PHB1: Prohibitin 1
SDHA: Succinate dehydrogenase complex flavoprotein subunit A
SNF: Solid not fat
SLC7A5: Solute carrier family 7 member 5
Thr: Threonine
Tau: Taurine
UQCRFS1: Ubiquinol-cytochrome c reductase, rieske iron-sulfur polypeptide 1
Val: Valine
VH: Villus height
VCR: Villus height to crypt depth ratio
Wnt/β-catenin: Wingless-related integration site/beta-catenin
