# Supplementary material for: Unraveling the effector mechanism of citrulline on sow lactation and offspring growth: an integrative multi-omics analysis
Source: J Anim Sci Biotechnol. 2026 Jun 16;17:122. doi: 10.1186/s40104-026-01414-x (PMC13270847; doi:10.1186/s40104-026-01414-x)
Supplement: Supplementary file 1 — Additional file 1: Table S1. Selection indicators of sampled sows. Table S2. Selection indicators of sampled suckling piglets. [file 40104_2026_1414_MOESM1_ESM.docx]

**Table S1** Selection indicators of sampled sows

| Treatments | Item | | | |
| --- | --- | --- | --- | --- |
|  | ADFI/kg | ADG of piglets/kg | Weight lose/kg | Weaning litter weight/kg |
| Con | | | | |
| Select | 6.83±0.56 | 218.89±6.71 | 7.67±5.91 | 64.49±5.38 |
| Total | 6.95±0.60 | 217.99±33.73 | 6.44±4.26 | 63.09±10.69 |
| *P*-value | 0.59 | 0.901 | 0.868 | 0.699 |
| Arg | | | | |
| Select | 6.53±1.90 | 239.15±17.40 | 6.56±18.95 | 70.23±6.19 |
| Total | 7.53±0.50 | 235.11±26.21 | 1.88±21.54 | 68.51±7.04 |
| *P*-value | 0.136 | 0.655 | 0.55 | 0.501 |
| 20%Cit | | | | |
| Select | 8.21±0.65 | 241.53±7.48 | 3.30±11.22 | 70.66±4.99 |
| Total | 8.06±0.82 | 246.87±8.39 | 4.36±14.54 | 68.95±11.11 |
| *P*-value | 0.607 | 0.545 | 0.836 | 0.643 |
| 30%Cit | | | | |
| Select | 7.90±0.85 | 234.64±14.75 | -15.10±19.37 | 67.16±4.92 |
| Total | 7.74±0.93 | 237.63±51.98 | -3.65±19.65 | 68.04±2.96 |
| *P*-value | 0.632 | 0.788 | 0.123 | 0.792 |
| 40%Cit | | | | |
| Select | 8.13±0.90 | 251.63±9.00 | -2.94±17.21 | 70.90±5.65 |
| Total | 7.94±0.74 | 245.44±38.78 | -9.61±24.63 | 69.24±10.49 |
| *P*-value | 0.528 | 0.444 | 0.437 | 0.639 |

*Select* Sows selected for sample collection in each group, *n*=10; *Total* The number of total experimental sows in each group, *n*=30.

Values are presented as mean ± standard deviation (SD).

**Table S2 The indices of the sampled piglets were analyzed.**

| Treatments | Item | |
| --- | --- | --- |
|  | weaning litter weight, kg | Weaning individual body weight of piglets |
| Con | | |
| Select | 64.37±11.15 | 5.87±0.27 |
| Total | 63.09±10.69 | 5.45±0.73 |
| *P*-value | 0.796 | 0.179 |
| Arg | | |
| Select | 66.44±5.79 | 5.52±0.38 |
| Total | 68.51±7.05 | 5.79±0.63 |
| *P*-value | 0.509 | 0.319 |
| 20%Cit | | |
| Select | 65.69±11.90 | 5.70±0.35 |
| Total | 68.94±11.11 | 5.96±0.94 |
| *P*-value | 0.526 | 0.507 |
| 30%Cit | | |
| Select | 74.32±11.61 | 5.97±0.19 |
| Total | 68.04±15.36 | 5.80±1.16 |
| *P*-value | 0.355 | 0.492 |
| 40%Cit | | |
| Select | 72.14±4.61 | 5.65±0.29 |
| Total | 69.24±10.49 | 5.97±0.88 |
| *P*-value | 0.515 | 0.396 |

Values are presented as mean ± standard deviation (SD).
