# Supplementary material for: Unraveling the effector mechanism of citrulline on sow lactation and offspring growth: an integrative multi-omics analysis
Source: J Anim Sci Biotechnol. 2026 Jun 16;17:122. doi: 10.1186/s40104-026-01414-x (PMC13270847; doi:10.1186/s40104-026-01414-x)
Supplement: Supplementary file 2 — Additional file 2: Fig. S1. Effects of maternal Cit supplementation on the expression of proteins related to mitochondrial metabolic function. Fig. S2. Effects of maternal Cit supplementation on the microorganisms in milk. Fig. S3. Effects of maternal 40%Cit on the metabolites in milk. [file 40104_2026_1414_MOESM2_ESM.docx]

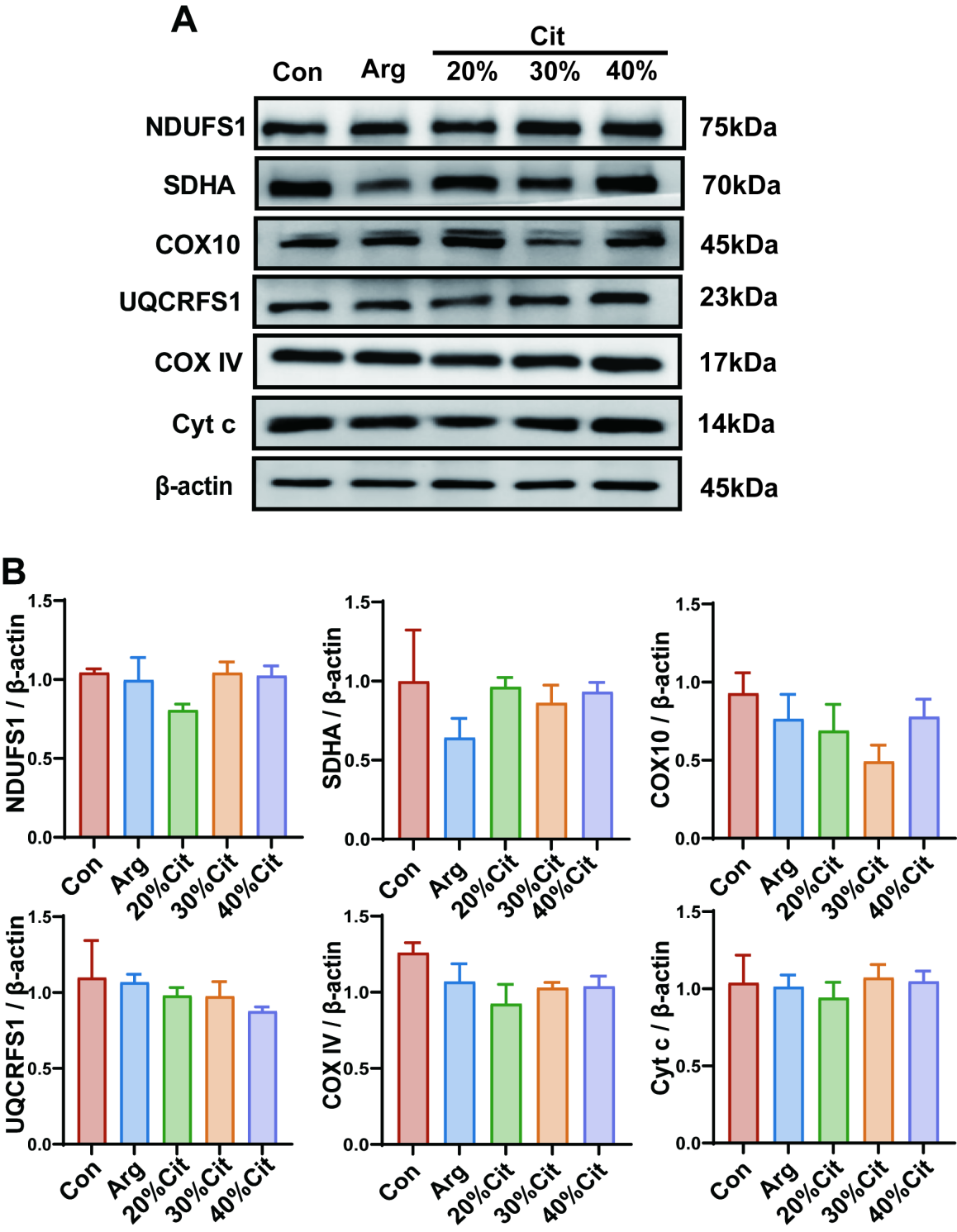


**Fig. S1** Effects of maternal Cit supplementation on the expression of proteins related to mitochondrial metabolic function. **A** and **B** The expression of mitochondrial metabolic functional proteins (*n* = 3)


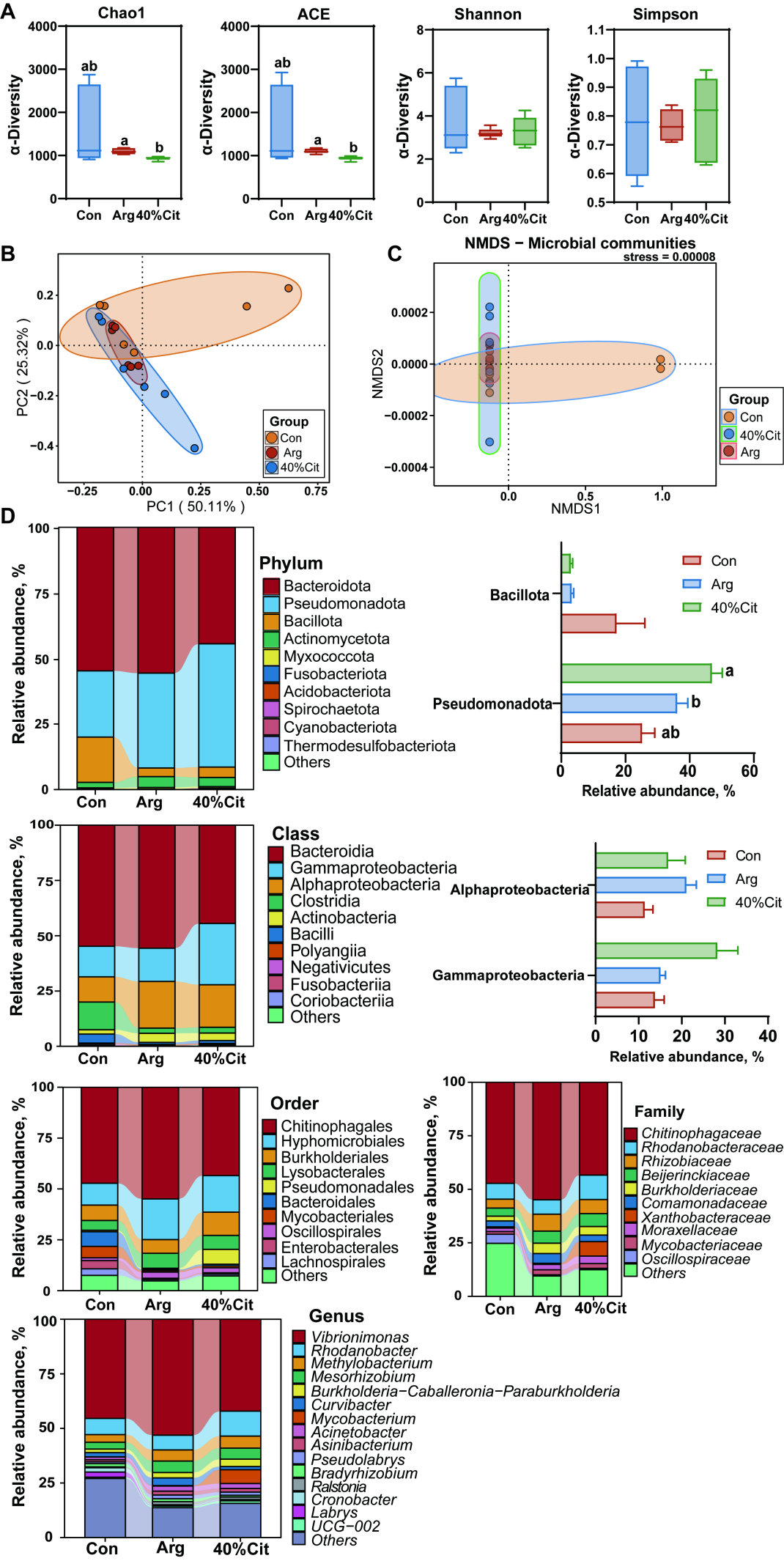


**Fig. S2** Effects of maternal Cit supplementation on the microorganisms in milk. **A** Results of α diversity analysis. **B** Principal coordinates analysis (PCoA) and non-metric multidimensional scaling (NMDS) plots showing the beta diversity of the microbiota in the samples. **C** Relative abundance of microbiota at different levels. Different letters (a, b) above peer data indicate significant differences (*P* < 0.05, *n* = 6)


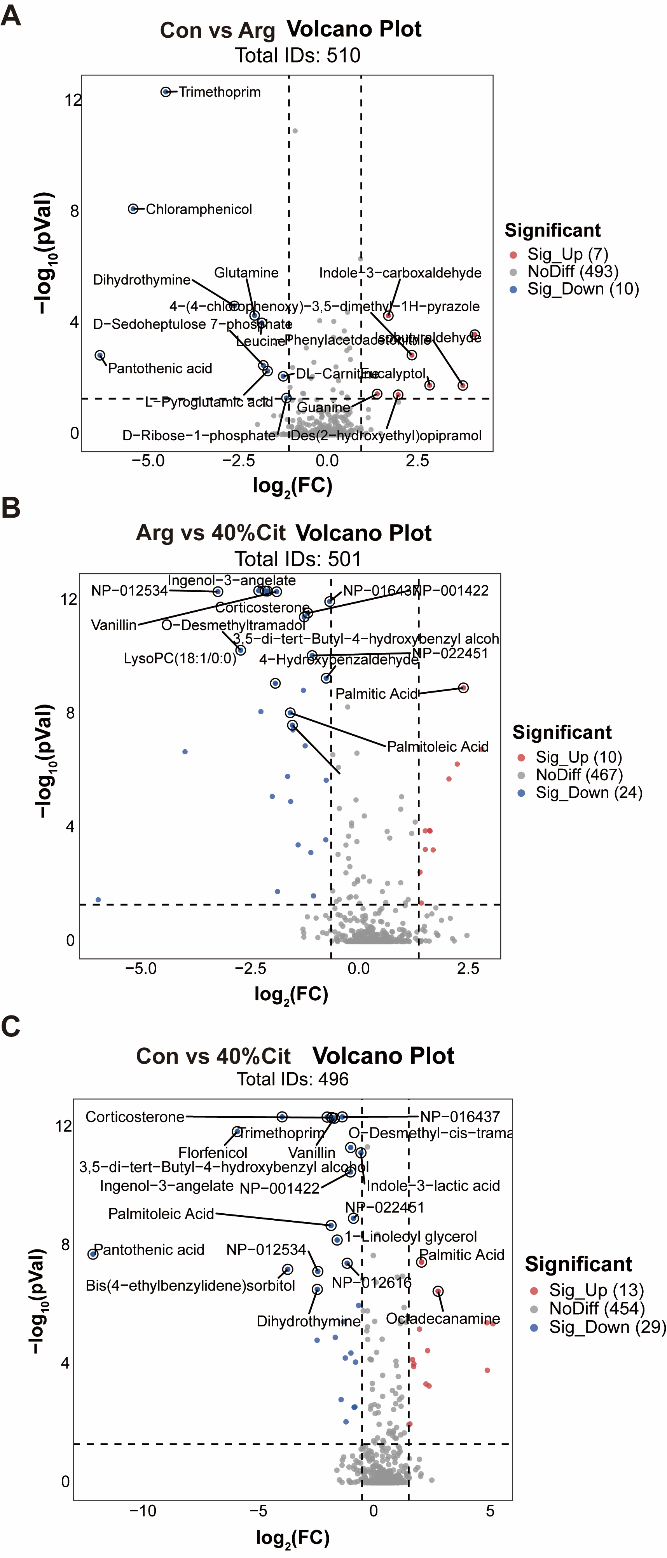


**Fig. S3** Effects of maternal 40%Cit on the metabolites in milk. **A**-**C** Volcano plot (Con vs Arg, Arg vs. 40%Cit and Con vs. 40%Cit). *n* = 6
