# Supplementary material for: Unraveling the effector mechanism of citrulline on sow lactation and offspring growth: an integrative multi-omics analysis
Source: J Anim Sci Biotechnol. 2026 Jun 16;17:122. doi: 10.1186/s40104-026-01414-x (PMC13270847; doi:10.1186/s40104-026-01414-x)
Supplement: Supplementary file 3 — Additional file 3. Original images for Western blot. [file 40104_2026_1414_MOESM3_ESM.docx]

Figure 1B

Occludin


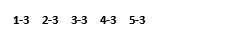

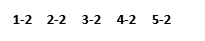

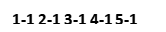

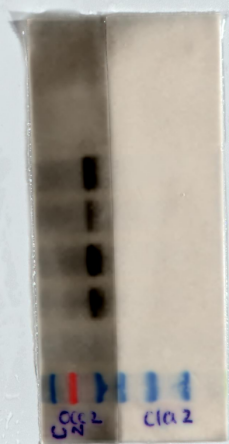

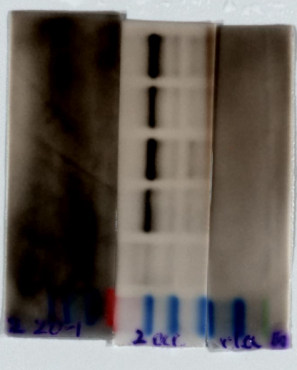

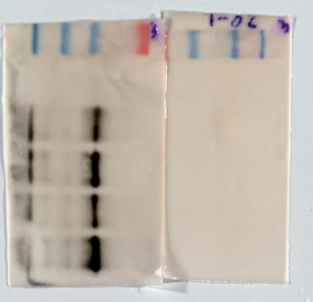


100KDa

75KDa

55KDa

45KDa

25KDa

35KDa


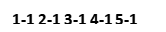

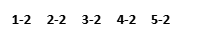
actin


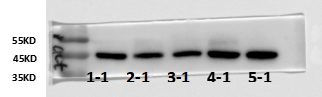

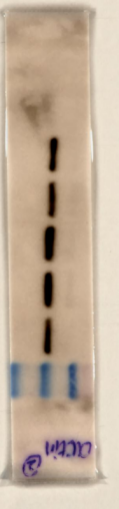


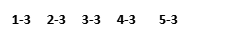

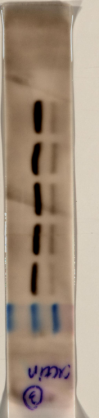


75KDa

55KDa

45KDa

Figure 1C

ZO-1


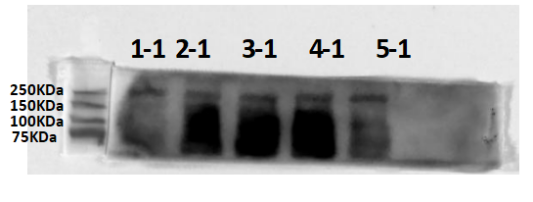


**1-2 1-3 2-2 2-3 3-2 3-3 4-2 4-3 5-2 5-3**


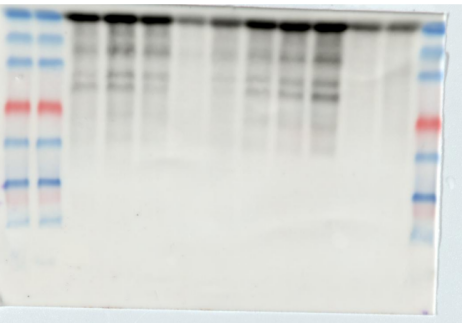

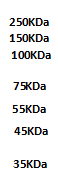


Claudin-1


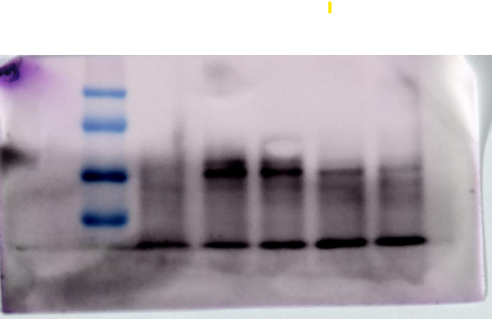


**1-2 1-3 2-2 2-3 3-2 3-3 4-2 4-3 5-2 5-3**


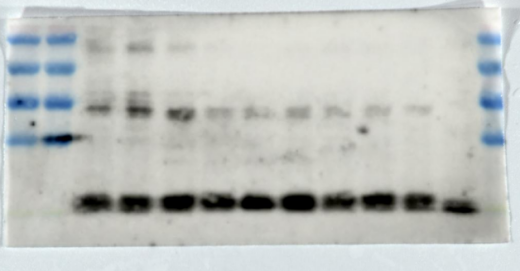


55KDa

45KDa

35KDa

14KDa

25KDa

1-1 2-1 3-1 4-1 5-1

actin

**1-2 1-3 2-2 2-3 3-2 3-3 4-2 4-3 5-2 5-3**


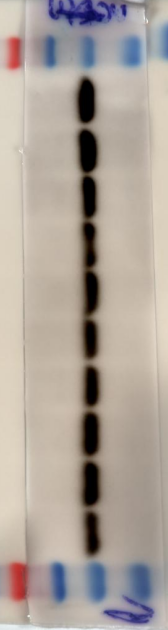


250KDa

150KDa

100KDa

55KDa

14KDa

45KDa

75KDa

25KDa


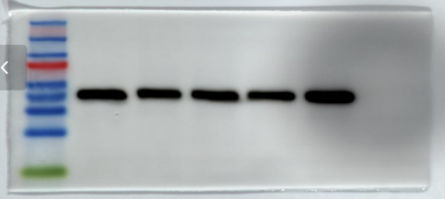


1-1 2-1 3-1 4-1 5-1

75KDa

55KDa

35KDa

45KDa

35KDa

Figure 2A

p-mTOR


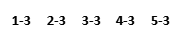

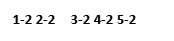

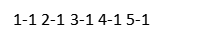


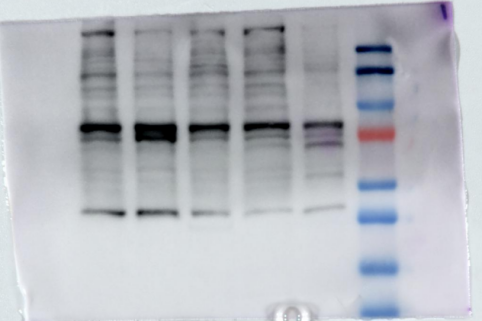

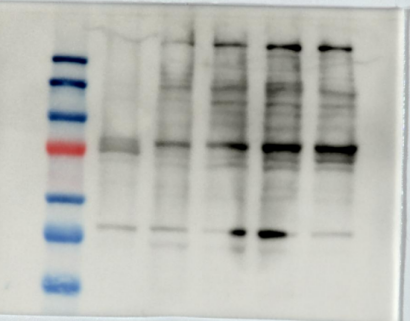


150KDa

100KDa

75KDa

55KDa

45KDa

35KDa

250KDa

150KDa

100KDa

75KDa

55KDa

45KDa

25KDa

35KDa

250KDa


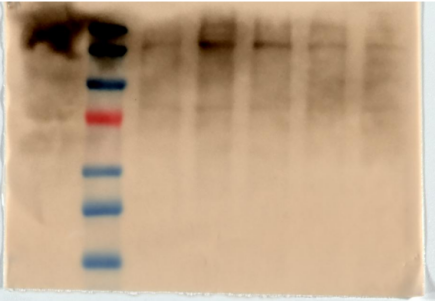


**35KDa**

**250KDa**

**45KDa**

**75KDa**

**55KDa**

**100KDa**


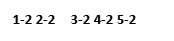
p-S6


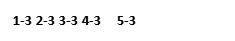

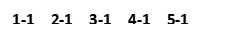

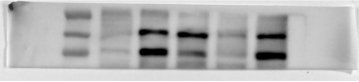

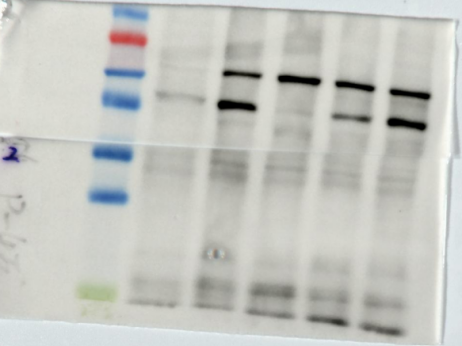


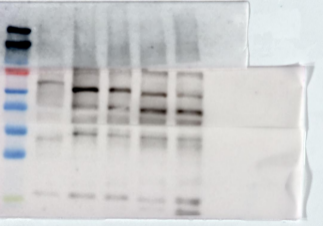

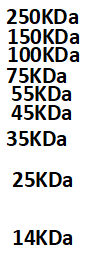


25KDa

100KDa

75KDa

55KDa

45KDa

35KDa

14KDa

75KDa

55KDa

45KDa


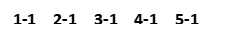
p-4EBP

1-2 2-2 3-2 4-2 5-2


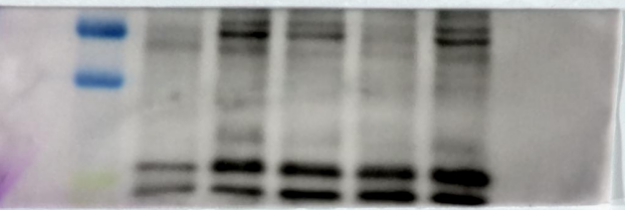

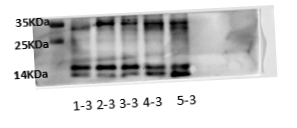


25KDa

35KDa

14KDa

25KDa

35KDa

14KDa


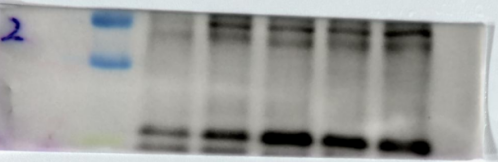


1-3 2-3 3-3 4-3 5-3

Actin


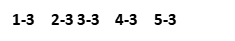

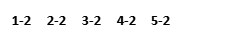

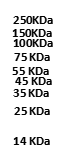

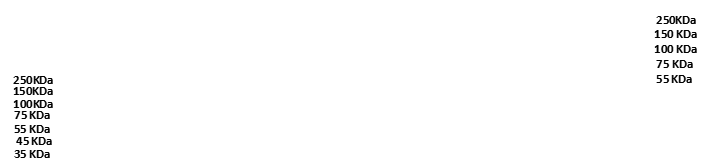

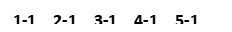

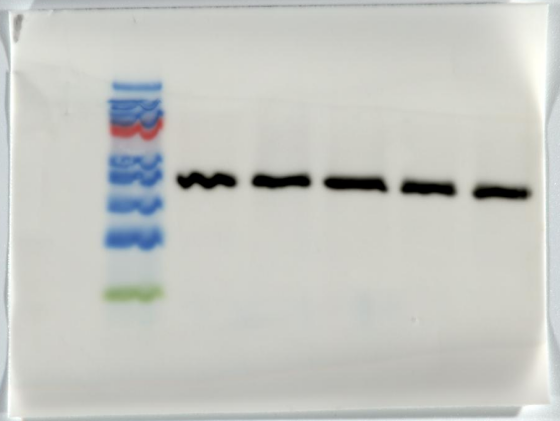

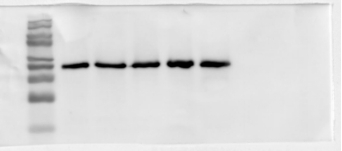

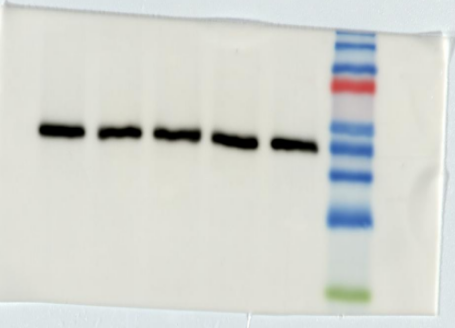


**25KDa**

**14KDa**

Figure 2C&2E

OPA-1

25KDa

100KDa

75KDa

55KDa

45KDa

35KDa

14KDa

150KDa

250KDa


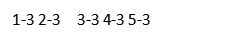

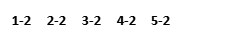

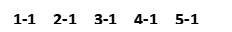

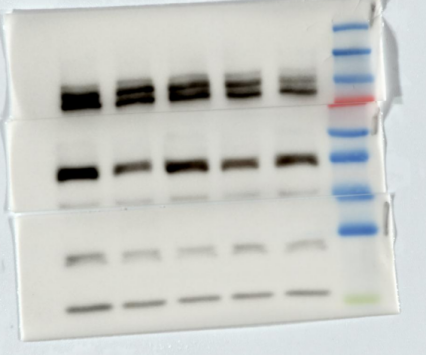

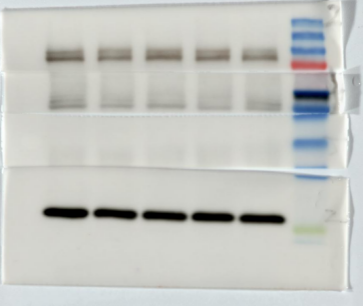


25KDa

100KDa

75KDa

55KDa

45KDa

35KDa

14KDa

150KDa

250KDa


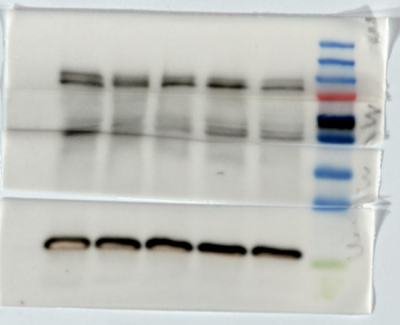

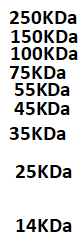


MFN2

1-1 2-1 3-1 4-1 5-1


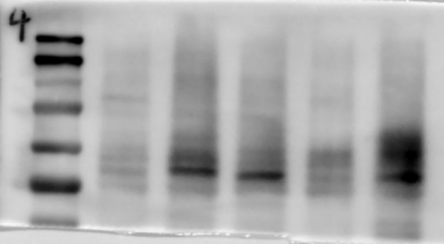

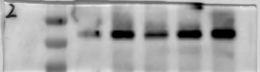


1-3 2-3 3-3 4-3 5-3

1-2 2-2 3-2 4-2 5-2

75KDa

55KDa

45KDa

100KDa

75KDa

55KDa

45KDa

35KDa

150KDa

250KDa


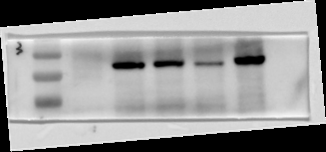


75KDa

55KDa

45KDa

PHB1

1-3 2-3 3-3 4-3 5-3

1-2 2-2 3-2 4-2 5-2

1-1 2-1 3-1 4-1 5-1

100KDa

75KDa

55KDa

45KDa

35KDa

150KDa

250KDa

25KDa


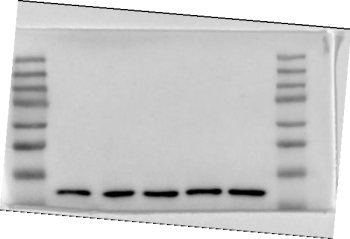

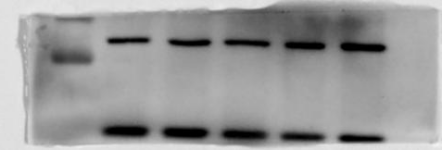

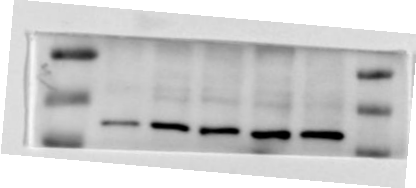


35KDa

25KDa

14KDa

35KDa

25KDa

14KDa

MFF

1-3 2-3 3-3 4-3 5-3

1-2 2-2 3-2 4-2 5-2

1-1 2-1 3-1 4-1 5-1

100KDa

75KDa

55KDa

45KDa

35KDa

150KDa

250KDa

25KDa

14KDa


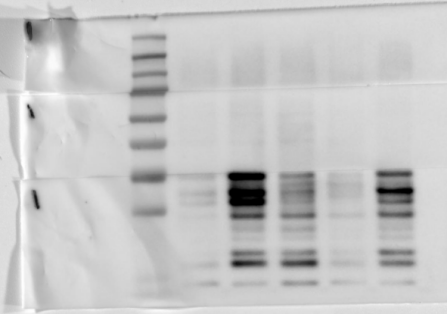

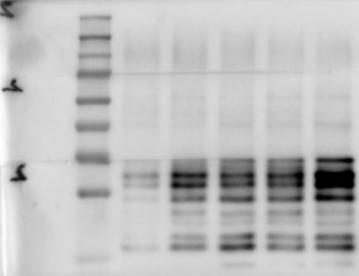

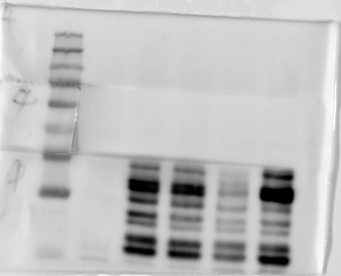


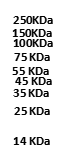
Actin

1-3 2-3 3-3 4-3 5-3

1-1 2-1 3-1 4-1 5-1


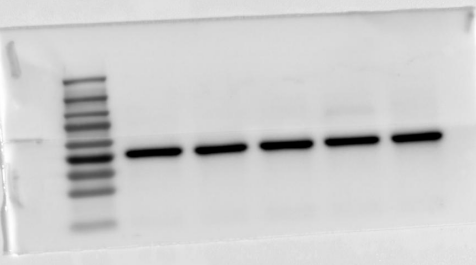

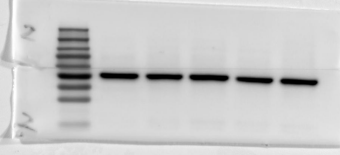

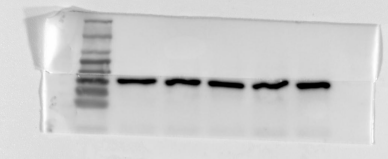


1-2 2-2 3-2 4-2 5-2

SLC7A5

1-2 2-2 3-2 4-2 5-2

1-1 2-1 3-1 4-1 5-1

75KDa

55KDa

45KDa


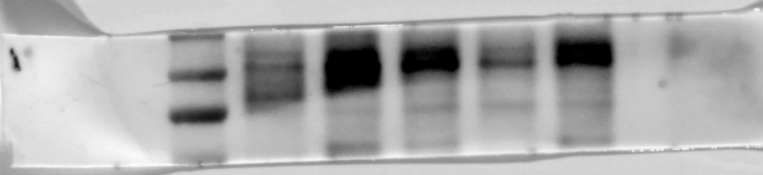

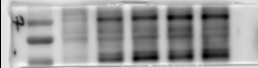


35KDa

55KDa

45KDa


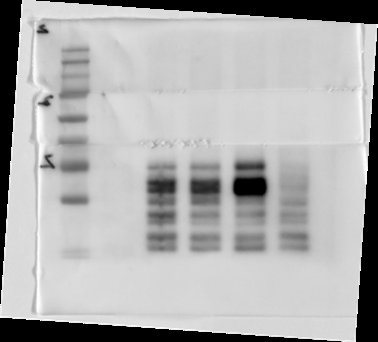


1-3 2-3 3-3 4-3 5-3

100KDa

75KDa

45KDa

55KDa

35KDa

150KDa

250KDa

25KDa

14KDa

Supplementary Figure1

NDUFSI

1-2 2-2 3-2 4-2 5-2

1-3 2-3 3-3 4-3 5-3

100KDa

75KDa

55KDa

45KDa

35KDa

150KDa

250KDa

25KDa

14KDa


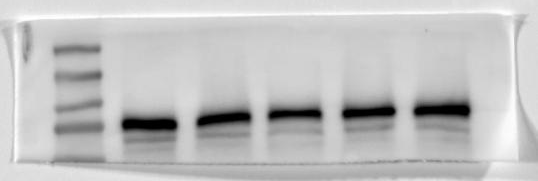

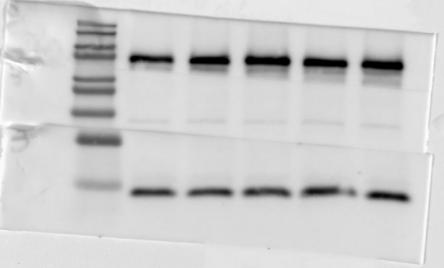

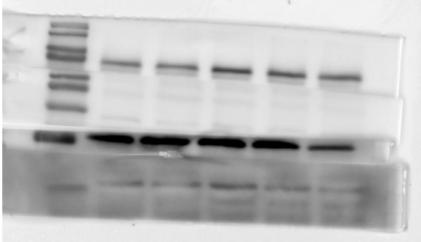


100KDa

75KDa

55KDa

45KDa

35KDa

150KDa

250KDa

25KDa

14KDa

1-1 2-1 3-1 4-1 5-1

100KDa

75KDa

55KDa

150KDa

250KDa

SDHA

1-3 2-3 3-3 4-3 5-3

1-2 2-2 3-2 4-2 5-2


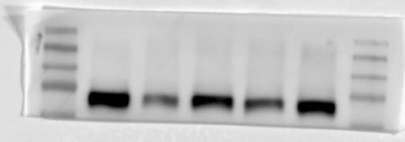

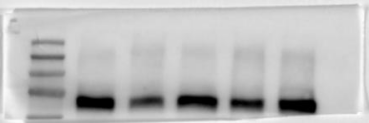

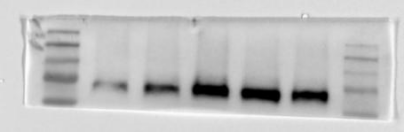


1-1 2-1 3-1 4-1 5-1

100KDa

250KDa

150KDa

75KDa

55KDa

COX10

1-3 2-3 3-3 4-3 5-3

35KDa

55KDa

45KDa

75KDa


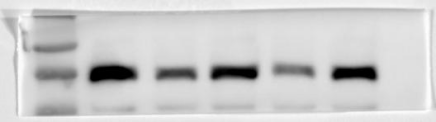

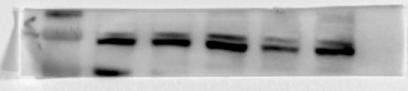

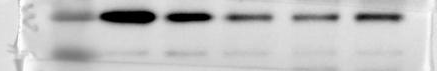


35KDa

45KDa

1-2 2-2 3-2 4-2 5-2

35KDa

55KDa

45KDa

1-1 2-1 3-1 4-1 5-1

UQCRFS1

1-3 2-3 3-3 4-3 5-3

1-2 2-2 3-2 4-2 5-2

1-1 2-1 3-1 4-1 5-1


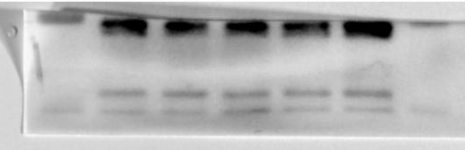

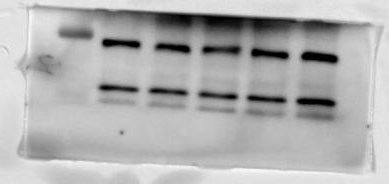

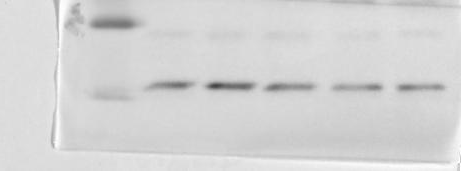


14KDa

25KDa

14KDa

25KDa

14KDa

25KDa

COX IV

14KDa

25KDa

1-3 2-3 3-3 4-3 5-3

14KDa

25KDa

1-2 2-2 3-2 4-2 5-2

1-1 2-1 3-1 4-1 5-1

14KDa

25KDa


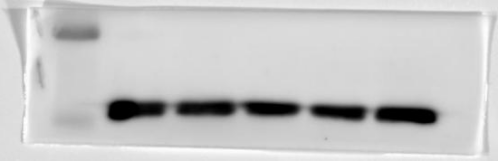

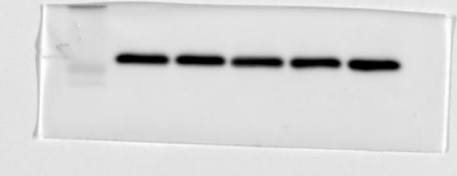

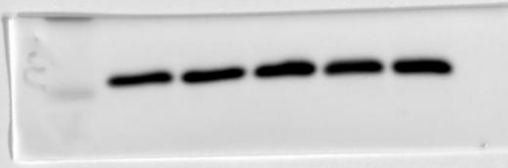


Cyt c


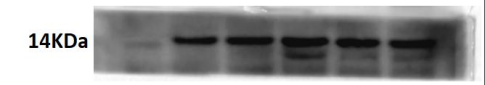

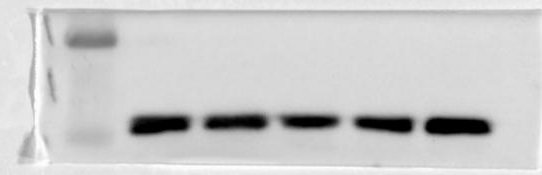

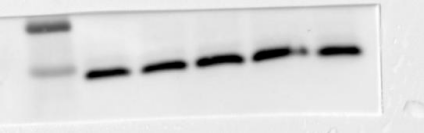


14KDa

25KDa

1-2 2-2 3-2 4-2 5-2

1-3 2-3 3-3 4-3 5-3

14KDa

25KDa

1-1 2-1 3-1 4-1 5-1

Actin

1-3 2-3 3-3 4-3 5-3


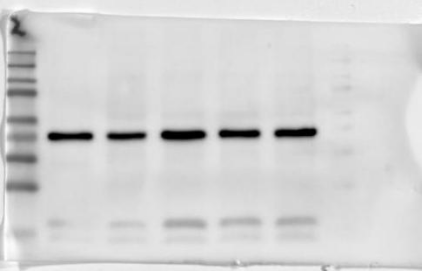

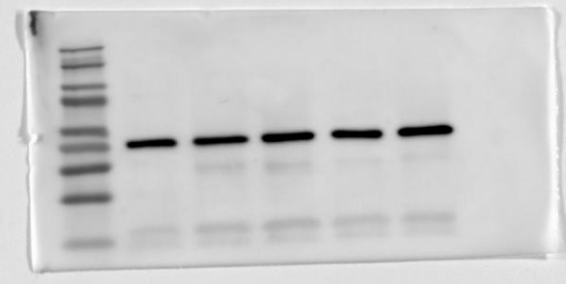

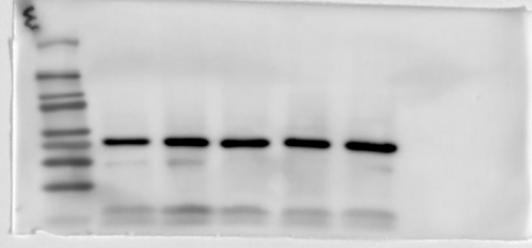


1-2 2-2 3-2 4-2 5-2

1-1 2-1 3-1 4-1 5-1

100KDa

75KDa

45KDa

55KDa

35KDa

150KDa

250KDa

25KDa

14KDa
